# Bayesian Geostatistical Model-Based Estimates of Soil-Transmitted Helminth Infection in Nigeria, Including Annual Deworming Requirements

**DOI:** 10.1371/journal.pntd.0003740

**Published:** 2015-04-24

**Authors:** Akinola S. Oluwole, Uwem F. Ekpo, Dimitrios-Alexios Karagiannis-Voules, Eniola M. Abe, Francisca O. Olamiju, Sunday Isiyaku, Chukwu Okoronkwo, Yisa Saka, Obiageli J. Nebe, Eka I. Braide, Chiedu F. Mafiana, Jürg Utzinger, Penelope Vounatsou

**Affiliations:** 1 Department of Biological Sciences, Federal University of Agriculture, Abeokuta, Nigeria; 2 Department of Epidemiology and Public Health, Swiss Tropical and Public Health Institute, Basel, Switzerland; 3 University of Basel, Basel, Switzerland; 4 Department of Zoology, Federal University, Lafia, Nigeria; 5 Mission to Save the Helpless (MITOSATH), Jos, Nigeria; 6 Sightsavers, Nigeria Country Office, Kaduna, Nigeria; 7 Department of Public Health, Federal Ministry of Health, Abuja, Nigeria; 8 National Universities Commission, Abuja, Nigeria; University of Minnesota, UNITED STATES

## Abstract

**Background:**

The acceleration of the control of soil-transmitted helminth (STH) infections in Nigeria, emphasizing preventive chemotherapy, has become imperative in light of the global fight against neglected tropical diseases. Predictive risk maps are an important tool to guide and support control activities.

**Methodology:**

STH infection prevalence data were obtained from surveys carried out in 2011 using standard protocols. Data were geo-referenced and collated in a nationwide, geographic information system database. Bayesian geostatistical models with remotely sensed environmental covariates and variable selection procedures were utilized to predict the spatial distribution of STH infections in Nigeria.

**Principal Findings:**

We found that hookworm, *Ascaris lumbricoides*, and *Trichuris trichiura* infections are endemic in 482 (86.8%), 305 (55.0%), and 55 (9.9%) locations, respectively. Hookworm and *A*. *lumbricoides* infection co-exist in 16 states, while the three species are co-endemic in 12 states. Overall, STHs are endemic in 20 of the 36 states of Nigeria, including the Federal Capital Territory of Abuja. The observed prevalence at endemic locations ranged from 1.7% to 51.7% for hookworm, from 1.6% to 77.8% for *A*. *lumbricoides*, and from 1.0% to 25.5% for *T*. *trichiura*. Model-based predictions ranged from 0.7% to 51.0% for hookworm, from 0.1% to 82.6% for *A*. *lumbricoides*, and from 0.0% to 18.5% for *T*. *trichiura*. Our models suggest that day land surface temperature and dense vegetation are important predictors of the spatial distribution of STH infection in Nigeria. In 2011, a total of 5.7 million (13.8%) school-aged children were predicted to be infected with STHs in Nigeria. Mass treatment at the local government area level for annual or bi-annual treatment of the school-aged population in Nigeria in 2011, based on World Health Organization prevalence thresholds, were estimated at 10.2 million tablets.

**Conclusions/Significance:**

The predictive risk maps and estimated deworming needs presented here will be helpful for escalating the control and spatial targeting of interventions against STH infections in Nigeria.

## Author Summary

Infections with three kinds of parasitic worms—hookworm, roundworm, and whipworm—are collectively known as soil-transmitted helminths (STHs). These parasitic worm infections are widespread in Nigeria, but the exact distribution is poorly understood. In view of the global commitment to control STH infections, there is a need to accelerate the mapping of STH infections to guide control interventions, such as large-scale administration of deworming drugs. In this study, we collated survey data from the year 2011 for Nigeria. The data were utilized to predict the distribution of STH infection based on environmental and socioeconomic covariates, and employing a Bayesian geostatistical modeling approach. Our results indicated that STH infections are widely distributed across Nigeria with prevalence estimates as high as 83% for roundworm, 50% for hookworm, and 19% for whipworm infections at specific survey locations. We predict that 5.7 million school-aged children were infected with STHs. The numbers of deworming tablets for annual or bi-annual treatment of the school-aged population at local government areas level in Nigeria for 2011 were estimated to be 10.2 million.

## Introduction

Soil-transmitted helminth (STH) infections belong to the neglected tropical diseases (NTDs). In terms of at-risk population and number of people infected, the STHs are the most frequent NTDs worldwide. The three common STHs are the roundworm (*Ascaris lumbricoides*), the whipworm (*Trichuris trichiura*), and the hookworms (*Ancylostoma duodenale* and *Necator americanus*) [1–3]. The most recent estimates suggest that 819 million people worldwide are infected with *A*. *lumbricoides*, 465 million with *T*. *trichiura*, and 439 million with hookworm [4]. STH infections thrive where there are poor hygiene practices, including limited environmental sanitation, unsafe water sources, inadequate toilet facilities, and poor fecal disposal methods, coupled with poverty and low household income [5–7]. School-aged children (5–14 years), in particular, are at high risk of infection and morbidity due to STHs, and hence, are the main target of preventive chemotherapy [8,9].

Nigeria has the highest total number of people infected with STHs in sub-Saharan Africa [10–12]. However, there is a paucity of empirical data on the spatial distribution of STH infections and this has hindered control. The planning, implementation, and rigorous monitoring of a national control program targeting STH infection can be enhanced with detailed knowledge of the spatial and temporal distribution of infection and morbidity [13]. In light of the global commitment to escalate the control of NTDs [14–17], knowledge of the spatial distribution of STH infections is a necessary prerequisite for the implementation of control and elimination measures, such as large-scale administration of anthelmintic drugs.

Thus far, two NTD-specific risk maps have been published for Nigeria; onchocerciasis [18] and schistosomiasis [19]. As the country prepares to implement large-scale preventive chemotherapy campaigns against STH infection, a nationwide map of the spatial distribution of STH using available survey data can help in advocacy, resource sourcing for funds, and implementation of control/elimination activities. The purpose of the current study was to produce high-resolution STH infection risk maps, including estimated number of school-aged children infected with *A*. *lumbricoides*, *T*. *trichiura*, and hookworm in Nigeria. We used recently obtained survey data and employed Bayesian geostatistical models to predict STH infection risk across Nigeria. Additionally, we computed annualized treatment requirements with the anthelmintic drugs albendazole and mebendazole. An important aspect of this study is to provide STH program managers with information for effective implementation of STH control activities.

## Methods

### Ethics Statement

The work presented here is derived from an in-depth analysis of STH infection survey data obtained from the Federal Ministry of Health (FMoH) of Nigeria in 2011. Ethical clearance, informed consent procedures, and treatment were according to FMoH guidelines and recommendations. The data are aggregated and do not contain identifiable individual or household level information. Thus, no specific ethical approval was required for the secondary analysis presented here.

### STH Infection Data

In 2011, a national survey was carried out in Nigeria, pertaining to STH infection among children aged 5–14 years. The overreaching goal of the survey was to prepare the country for mass drug administration with albendazole or mebendazole, provide evidence-based data for advocacy, funding, and support of preventive chemotherapy. The survey was conducted by FMoH, in collaboration with State Ministries of Health and non-governmental organizations using trained field workers. The survey used standard protocols put forth by the World Health Organization (WHO) for rapid mapping of STH infection in schools, collection of stool samples, and laboratory work-up. The diagnostic method used was the Kato-Katz technique, with duplicate Kato-Katz thick smears prepared from fresh stool samples; one per participant [20]. In each community/school, 60 school-aged children were examined. Data were collected at 555 locations across Nigeria, with the exception of areas in the north-eastern part of the country, where the state of security at the time of the survey did not allow doing so. Study locations were geo-referenced, using a hand-held global positioning system (GPS) device (Garmin Etex; Garmin Corp, Kansas, United States of America). Quality checks were performed to authenticate that the coordinates indeed corresponded to specific locations using readily available Google Map and Google Earth tools.

### Environmental Data

Environmental data were obtained from open-access remote sensing data sources, as detailed in Table 1. These include normalized difference vegetation index (NDVI) as vegetation proxy, day and night land surface temperature (LST), altitude, soil acidity, and soil moisture. Environmental data were processed as described elsewhere [21]. Annual averages for the year 2011 were calculated and used in all subsequent analyses. Maps showing the variation of the covariates across the country are shown in the Supporting Information (S2 Fig).

**Table 1:** Sources of environmental and socioeconomic data used to model soil-transmitted helminth infection risk in Nigeria.

Data type	Source	Date	Temporal resolution	Spatial resolution
Altitude	SRTM^1^	2011		1 km
Rainfall	FEWS NET^2^	2011	Yearly	1 km
Normalized difference vegetation index	MODIS/Terra^3^	2009–2011	16 days	1 km
Day land surface temperature	MODIS/Terra^3^	2009–2011	8 days	1 km
Night land surface temperature	MODIS/Terra^3^	2009–2011	8 days	1 km
Soil pH/soil moisture	ISRIC-WISE^4^			10 km
Population density	Afripop^5^	2010		1 km
Urban-rural classification	SEDAC^6^			1 km

^1^ Shuttle Radar Topography Mission (SRTM); http://www.worldclim.org/ (accessed on 20 July 2014).

^2^ Famine Early Warning System (FEWS) Network; http://earlywarning.usgs.gov/fews/index.php/ (accessed on 20 July 2014).

^3^ Moderate Resolution Imaging Spectroradiometer (MODIS); https://lpdaac.usgs.gov/ (accessed on 20 July 2014).

^4^ Global soil profile data ISRIC-WISE database v.1.2; http://www.isric.org/ (accessed on 20 July 2014).

^5^ World Population database; http://www.clas.ufl.edu/users/atatem/index_files/Nigeria.htm (accessed on 20 July 2014).

^6^ Socioeconomic Data and Applications Center; http://sedac.ciesin.columbia.edu/data/dataset/grump-v1-urban-extents (accessed on 20 July 2014).

### Socioeconomic and Population Data

Data on rural and urban extents for Nigeria were downloaded from the Center for International Earth Science Information Network [22]. Population data for 2010, at 100 x 100 m spatial resolution, was downloaded from the Afripop population database hosted by the World Population. The data were adjusted to 2011 by multiplying each pixel value with the Nigerian annual growth rate of 2.8% (http://data.worldbank.org/indicator/SP.POP.GROW). The total population for 2011 was 154,731,365 of which 26.8% correspond to school-aged children (http://www.census.gov/population/international/data/idb/region.php).

### Statistical Analysis

We applied Bayesian binomial geostatistical models to relate STH infection risk with environmental and socioeconomic predictors. We used integrated nested Laplace approximations (INLA) [23] and a stochastic partial differential equations approach [24] for fast approximate Bayesian inference. Analysis was carried out in R [25] and the INLA package (www.r-inla.org). Details of how models were implemented are provided in Supporting Information (S1 Text) [24,26,27].

We followed an approach detailed by Karagiannis-Voules *et al*. [28], which has also been used for STH geostatistical modeling in Cambodia [29], to select the best predictive model. In brief, we fitted Bayesian bivariate geostatistical models to select the functional form of the effect of each predictor based on the cross-validated logarithmic score [30,31]. We considered linear and categorical functional forms of effects. The categorical functional form of the covariates was generated using 25^th^, 50^th^, and 75^th^ percentile to group each covariate into specific categories. Non-linearity was addressed through random walk processes of order 1 and 2 [32]. The form of each predictor giving the lowest mean logarithmic score was chosen. To identify the set of the most important predictors, we fitted geostatistical models with all possible combination of covariates (i.e., 256 models for each STH species-specific infection) and selected the one, for each of the three STH species, with the best logarithmic score. The final models were used to predict infection risk at a grid of 3 x 3 km including areas where infection data were not available. The form of the covariate that was included in the final model used in the prediction of each species of STH is shown in Table 2. The posterior estimates and Bayesian credible intervals for the effects of the predictors are presented in odds ratios. Additional details are provided in Supporting Information (S1 Text).

**Table 2:** Posterior estimates (median; 95% Bayesian credible interval) of the final geostatistical models for soil-transmitted helminth infections in Nigeria in 2011.

Species	Predictor variable	Median (95% Bayesian credible interval)
*Ascaris lumbricoides*	NDVI 2011	1.29 (1.00, 1.66)
	Altitude (m)	
	<186	1.00
	186–305	0.71 (0.40, 1.25)
	≥305	1.50 (0.79, 2.77)
	LST day 2011 (°C)	
	<29.5	1.00
	29.5–34.0	0.56 (0.36, 0.87)
	≥34.0	0.13 (0.06, 0.27)
Hookworm	Rural	1.00
	Urban	0.79 (0.63, 1.00)
	NDVI 2011	
	<0.28	1.00
	0.28–0.40	1.02 (0.84, 1.23)
	0.40–0.48	1.30 (1.00, 1.68)
	≥0.48	1.67 (1.25, 2.22)
	LST day 2011 (°C)	
	<29.5	1.00
	29.5–34.0	0.77 (0.59, 1.01)
	≥ 34.0	0.88 (0.60, 1.28)
*Trichuris trichiura* *	Altitude (m)	
	<420.5	1.00
	420.5–835	0.11 (0.03, 0.33)
	835–1,249	0.65 (0.26, 1.64)
	≥1,249	0.30 (0.10, 0.93)

*The effect of land surface temperature (LST) at night is depicted in S1 Fig.

Due to the large number of observed zero prevalence data, we additionally fitted zero-inflated binomial models with invariant probability of zero-inflation. These models have shown better predictive ability in geostatistical modeling of malaria. [33]. In the present study, the zero-inflated models did not improve predictions (based on the cross-validated logarithmic score). Hence, we report results from the binomial models.

### Determination of School-Aged Population at Risk of STH

The school-aged population infected with STHs was estimated by combining the predictive posterior distribution of the infection prevalence at the pixel level with the school-aged population size at each pixel. The number of infected school-aged children was calculated by summing the respective values for each pixel, as described by Schur *et al*. [34].

### Estimation of Anthelmintic Treatment Needs

The amount of anthelmintic treatment (i.e., albendazole or mebendazole) that would be required to treat infected school-aged children at the unit of the state in Nigeria was computed from the pixel level risk estimates. Following recommendations by WHO, school-aged children should be treated twice a year in areas where the infection prevalence is ≥50%, while annual treatment is recommended in areas where infection prevalence ranges between 20% and 50% [9]. Hence, we computed the total number of anthelmintic drugs needed by multiplying the number of school-aged children, per pixel, by a factor of 2 (prevalence ≥50%, biannual treatment) or 1 (prevalence 20–50%, annual treatment). We considered the estimated prevalence of STH at pixel-level, calculated under the assumption that the species-specific prevalences are independent. Treatments were aggregated over all pixels within individual states [19,33]. We compared treatment needs calculated from both pixel and population-adjusted district level prevalences. The estimation of the country-wide number of treatments was based on the sum of the treatment distributions of all local government areas (LGAs) and was conducted using both the approaches described above.

## Results

### Spatial Distribution of STH Infections in Nigeria

**Fig 1:**
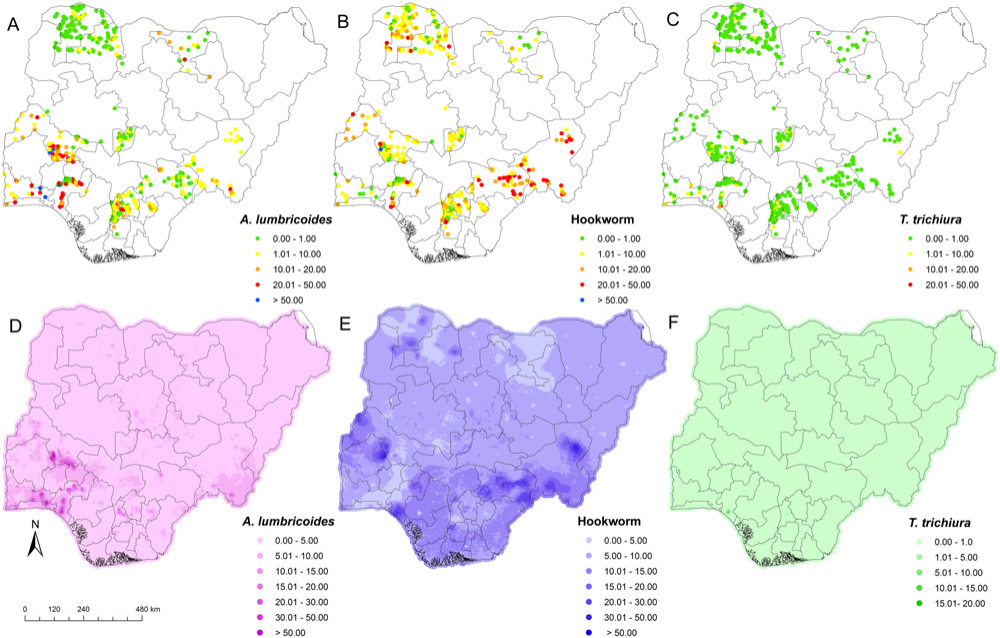
Spatial distribution of soil-transmitted helminth infections in Nigeria. A) Observed prevalence of *A*. *lumbricoides*. B) Observed prevalence of hookworm. C) Observed prevalence of *T*. *trichiura*. D) Predicted prevalence of *A*. *lumbricoides*. E) Predicted prevalence of hookworm. F) Predicted prevalence of *T*. *trichiura*.

STH infections were diagnosed in the stool of school-aged children surveyed in 20 of the 36 states, including the Federal Capital Territory, Abuja. *A*. *lumbricoides* was present in 305 (55.0%) locations in 16 states, and prevalence at the unit of the state varied from 1.6% to 77.8% (Fig 1A). Hookworm infection showed the widest geographic distribution, as it was found in 482 (86.8%) locations in all 20 states, with prevalence at the unit of the state ranging from 1.7% to 51.7% denoted with the varying colours in Fig 1B. *T*. *trichiura* was found in 55 (9.9%) locations in 12 states with state-prevalence ranging from 1.0% to 25.5% (Fig 1C). *A*. *lumbricoides* and hookworm HHhHhinfections were co-endemic in 16 states, while co-occurrence of all three STH species was observed in 12 states.

### Predicted Risk of *A*. *lumbricoides*

Areas with high infection risk (≥50%) of *A*. *lumbricoides* were predicted for the south-western part of Nigeria. For most areas in the northern and southern parts of Nigeria, the predicted prevalence was below 5% (Fig 1D). Predicted pixel level prevalence revealed that high risks areas for *A*. *lumbricoides* infection occur within the states of Ogun, Ondo, Kwara, and Kogi, and some areas in Anambra and Taraba states. Our Bayesian geostatistical model for *A*. *lumbricoides* risk suggests that extreme high LST (≥34°C) is negatively associated with *A*. *lumbricoides*, while a positive association was found between *A*. *lumbricoides* infection and high NDVI value (Table 2).

### Predicted Risk of Hookworm

It was observed that most south-eastern, western, and middle belt parts of Nigeria fell either in the high-risk (≥50% pixel level prevalence) or moderate-risk areas (20–50% pixel level prevalence) of hookworm infection (Fig 1E). The predicted pixel level risk of hookworm in the northern states of Nigeria ranged from 5% to 10% (Fig 1E). However, there are some high-risk (≥50% pixel level prevalence) communities in the states of Katsina, Zamfara, and Sokoto in the north-western part of the country. Only few areas in Nigeria showed a pixel level predicted prevalence of hookworm below 5%. Areas with predicted hookworm pixel level prevalence greater than ≥50%, which are considered as high-risk areas, were observed in the states of Taraba, Benue, Oyo, Kwara, Katsina, Zamfara, Sokoto, and Kebbi (Fig 1E). The risk of hookworm infection at pixel level in Jigawa, Ogun, Osun, and parts of Zamfara and Sokoto states were predicted to be below 5%. Our Bayesian-based geostatistical model for hookworm showed that high NDVI values and low day LST values are positively associated with hookworm infection (Table 2). The prevalence of hookworm infection was lower in urban compared to rural areas (Table 2).

### Predicted Risk of *T*. *trichiura*

Infection risk of *T*. *trichiura*, ranging between 10% and 20% (pixel level prevalence), was predicted for areas in the south-west (Ondo state), while all other parts of the country showed pixel level prevalence risks below 10%. The predicted risk of *T*. *trichiura* was considerably higher in the southern part of Nigeria compared to the north (Fig 1F). Pixel level prevalence revealed that areas within Ogun, Ondo, Anambra, and Enugu states and some areas of Taraba state are at high risk of *T*. *trichiura*. Our Bayesian geostatistical model for *T*. *trichiura* suggests a random walk process for night LST, indicating that extreme high temperatures (≥34°C) are associated with the absence of *T*. *trichiura* in Nigeria. A negative association was found between altitude (increase in altitude) and risk of *T*. *trichiura* infection.

### School-Aged Population at Risk of STHs and Treatment Requirements

Out of the 41.5 million school-aged children in Nigeria, an estimated 5.7million are predicted to be infected with any STH, an overall predicted prevalence of 13.8%. Kano state has the highest number of infected school-aged children, while the Federal Capital Territory, Abuja has the lowest prevalence (Table 3).

**Table 3:** Median predicted prevalence of soil-transmitted helminth infection; number of school-aged children infected for Nigeria, stratified by state for 2011.

		Population-adjusted mean prevalence and 95% Bayesian credible interval				
S/N	State	*Ascaris lumbricoides* (95% CI)	*Trichuris trichiura* (95% CI)	Hookworm (95% CI)	Any STH**	School-aged children population infected
1	Abia	7.8 (2.8, 19.7)	0.3 (0.1, 0.7)	9.3 (3.5, 20.3)	16.8 (8.3, 30.0)	144,950
2	Adamawa	2.8 (1.6, 4.2)	0.1 (0.1, 0.2)	8.5 (5.1, 13.4)	11.2 (7.6, 16.9)	92,727
3	Akwa Ibom	8.3 (3.1, 19.4)	0.4 (0.1, 1)	9.5 (3.2, 23.4)	18.4 (8.9, 31.3)	199,303
4	Anambra	3.9 (2.7, 6.2)	0.2 (0.1, 0.4)	6.3 (5.5, 7.5)	10.2 (8.5, 12.9)	126,859
5	Bauchi	3.6 (2.2, 5.9)	0.1 (0.04, 0.2)	7.3 (4.5, 10.1)	10.7 (7.6, 14.3)	145,923
6	Bayelsa	8.0 (4.0, 17.6)	0.3 (0.1, 0.9)	11.2 (4.3, 24.0)	19.2 (11.4, 32.7)	83,072
7	Benue	5.2 (3.7, 7.7)	0.2 (0.1, 0.3)	14.8 (12.4, 18.2)	19.7 (16.7, 23.6)	247,591
8	Borno	1.8 (0.9, 4.3)	0.1 (0.1, 0.3)	7.2 (4.5, 12.2)	9.2 (6.46, 13.4)	108,514
9	Cross River	8.9 (5.1, 14.0)	0.3 (0.1, 0.7)	12.0 (7.4, 18.9)	20.4 (13.8, 28.3)	155,420
10	Delta	7.7 (3.9, 14.4)	0.3 (0.1, 0.8)	10.8 (5.4, 19.6)	18.1 (12.0, 27.3)	211,644
11	Ebonyi	7.6 (3.7, 15.5)	0.3 (0.1, 0.8)	7.8 (4.3, 13.9)	15.5 (9.6, 22.8)	94,252
12	Edo	7.6 (4.1, 15.2)	0.2 (0.1, 0.8)	10.2 (6.3, 17.4)	17.9 (11.9, 27.6)	175,147
13	Ekiti	10.4 (3.9,21.7)	0.1 (0.02, 0.2)	7.6 (3.8, 14.3)	17.8 (10.3, 28.9)	120,899
14	Enugu	6.4 (3.5, 14.2)	0.2 (0.1, 0.4)	7.9 (6.1, 10.1)	14.2 (10.4, 21.8)	135,568
15	Federal Capital Territory	4.2 (2.5, 7.6)	0.3 (0.2, 0.5)	7.5 (5.8, 9.6)	11.6 (9.2, 15.3)	43,500
16	Gombe	2.2 (1.1, 4.5)	0.1 (0.1, 0.3)	7.0 (4.3, 13.9)	9.4 (6.3, 15.8)	67,031
17	Imo	6.3 (2.5, 16.3)	0.2 (0.1, 0.5)	12.3 (5.2, 23.8)	18.5 (10.6, 29.9)	210,835
18	Jigawa	3.4 (1.7, 5.2)	0.1 (0.02, 0.2)	5.0 (3.3, 7.1)	8.1 (5.9, 11.3)	105,754
19	Kaduna	7.2 (4.4, 11.5)	0.04 (0.02, 0.2)	7.5 (4.8, 12.1)	14.8 (10.7, 19.7)	265,200
20	Kano	5.0 (2.5, 12.3)	0.1 (0.01, 0.3)	5.8 (3.0, 14.6)	11.6 (6.4, 20.3)	322,757
21	Katsina	4.2 (2.4, 7.7)	0.04 (0.01, 0.2)	7.1 (4.2, 10.8)	11.0 (7.4, 16.6)	185,648
22	Kebbi	1.9 (1, 3.2)	0.1 (0.04, 0.1)	7.8 (4.7, 11.9)	9.9 (6.6, 14.0)	95,208
23	Kogi	8.2 (4.7, 12.7)	0.2 (0.1, 0.4)	10.6 (6.7, 16.6)	18.1 (13.4, 25.1)	178,799
24	Kwara	20.9 (17.1, 24.7)	0.2 (0.1, 0.6)	7.7 (6.7, 8.9)	27.3 (23.6, 30.8)	199,369
25	Lagos	5.9 (1.4, 27.0)	0.2 (0.1, 2.1)	3.1 (1.1, 9.7)	10.4 (4.4, 28.4)	291,630
26	Nassarawa	5.7 (3.5, 9.7)	0.14 (0.1, 0.3)	8.6 (5.0, 14.4)	14.2 (9.6, 20.3)	79,619
27	Niger	4.6 (3.2, 7.4)	0.2 (0.1, 0.3)	7.8 (5.4, 10.4)	12.5 (9.4, 16.2)	142,791
28	Ogun	11.8 (7.4, 17.3)	0.4 (0.2, 1.0)	5.1 (3, 8.5)	16.8 (11.9, 22.2)	173,848
29	Ondo	13.5 (9.7, 18.0)	0.6 (0.4, 1.2)	11.6 (8.4, 17.0)	23.9 (18.6, 29.6)	219,037
30	Osun	8.5 (3.7, 15.8)	0.1 (0.1, 0.3)	4.5 (2.2, 9.3)	12.8 (7.9, 20.1)	143,953
31	Oyo	6.5 (3.6, 13.5)	0.1 (0.1, 0.3)	7.7 (4.8, 13.5)	14.0 (9.4, 21.6)	236,550
32	Plateau	5.9 (3.2, 11.9)	0.1 (0.03, 0.4)	7.8 (4.3, 12.7)	14.0 (8.9, 19.4)	131,414
33	Rivers	7.9 (2.9, 19.8)	0.3 (0.1, 1.1)	10.0 (4.3, 20.9)	18.3 (9.5, 31.6)	221,281
34	Sokoto	1.4 (0.9, 2.0)	0.2 (0.1, 0.4)	6.8 (6.0, 7.9)	8.3 (7.5, 9.7)	88,095
35	Taraba	6.1 (4.6, 7.9)	0.1 (0.1, 0.3)	14.1 (11.3, 16.8)	19.6 (16.4, 22.3)	132,344
36	Yobe	2.0 (0.9, 3.9)	0.1 (0.1, 0.2)	6.6 (3.9, 11)	8.7 (6.1, 12.9)	59,787
37	Zamfara	3.3 (2.3, 4.9)	0.1 (0.1, 0.2)	7.3 (5.8, 9.2)	10.5 (8.9, 12.5)	100,545
	Total/Mean	6.2 (3.3, 12.5)	0.17 (0.08,0.55)	7.9 (4.8, 13.5)	13.8 (10.5, 16.5)	5,736,864

**Calculated under the assumption of independence.

Following WHO recommended cut-offs of 20% and 50% for annual and bi-annual preventive chemotherapy with either albendazole or mebendazole, the estimates aggregated at state level showed that only 3 out of the 37 states had a population-adjusted prevalence between 20% and 50%. These states are Cross River, Kwara, and Ondo (Table 3). The LGA is the third administrative level in Nigeria and the preferred unit for health intervention. According to the aforementioned prevalence cut-offs, we computed that the number of albendazole or mebendazole tablets needed for treatments using pixel-level prevalence is 10,222,409, tablets, whereas using population-adjusted LGA-level prevalence, it is 9,025,229 tablets. These numbers correspond to the median of the country-wide distributions of treatment needs rather than the sum of the median LGA predicted requirements (S1 Table).

## Discussion

We provide spatially explicit model-based risk estimates of the three main species of STHs in Nigeria. We used Bayesian geostatistical methods which have become essential tools in infectious disease risk profiling [35]. Our estimates are based on a large ensemble of recent survey data that were obtained using standard protocols. Hence, our estimates are more robust than those obtained from previous mapping exercises that collated historic survey data employing different collection methods and diagnostic approaches [28,36,37]. Our predictive risk maps are important and useful for planning, implementation, and evaluation of STH control programs [21]. Indeed, as a first step, the maps will help prioritize the implementation of intervention programs for the control of STH infections, particularly the spatial targeting of preventive chemotherapy. This is important in light of the current global moves toward control and elimination of NTDs [14,38]. Additionally, the model-based risk map of STH presented here complements a recent model-based risk map of schistosomiasis in Nigeria [19] for concurrent control of STH and schistosomiasis [39,40]. An integrated approach for the control of multiple helminthiases would reduce operational costs in the planning and implementation of control programs, as the primary target risk group for preventive chemotherapy are school-aged children, and hence the education system is the most convenient platform for drug administration [38,41,42]. It should be noted, however, that recent mathematical modeling work revealed that adults should also be targeted by preventive chemotherapy if substantial gains of morbidity control and interruption of transmission are aimed for [43]. A similar result was supported by a sub-continental geostatistical analysis of STH in sub-Saharan Africa [28].

Our predictions show that most areas in Nigeria are characterized by STH infection prevalence below 20%. This estimate is in line with the distribution pattern of STH infections in most endemic populations. Infections are usually aggregated where most infected individuals in a community will have infections of light or moderate intensity, while a few will be heavily infected [44]. The heavily infected individuals are at highest risk of clinical consequences of STH infection and serve as the reservoir host for the continuous transmission to the rest of the community [41]. Although WHO does not recommend large-scale preventive chemotherapy in areas where prevalence is below 20% [9], detailed information of the number of infected individuals for lower risk areas is important from operational and programmatic points of view [45]. The overall relatively low prevalence of STH infection across Nigeria could be due to the periodic deworming of school-aged children by health officials and non-governmental health organizations working in the country. Currently ongoing in Nigeria are deworming programs targeting onchocerciasis and lymphatic filariasis, which include ivermectin treatment given to school-aged children 5 years and above for onchocerciasis and/or ivermectin plus albendazole against lymphatic filariasis. Another reason may be attributed to good access to cheap sachet drinking water popularly called “pure water” in many rural communities in Nigeria. This 500 ml nylon-bagged potable water is basically available everywhere in Nigeria and is sold at US$ 0.03 per sachet. The availability of this product may be a factor in reducing the fecal-oral transmission of *A*. *lumbricoides* and *T*. *trichiura*. On the other hand, the comparatively higher prevalence and distribution of hookworm infection in Nigeria is associated with the transmission of this parasite through the skin. Hence, barefoot walking by school-aged children is a risk factor and is likely to be driven by low socioeconomic status [44]. It should also be noted that Nigeria in the equatorial zone is suitable for hookworm larval development [46].

Our predictions revealed that less than 15% of school-aged children were infected with STHs in 2011. Thus, the acceleration of STH control is important to maintain this relatively low level of prevalence in the most populous country in Africa [47]. Our data are useful in reviewing the current STH control program in Nigeria in light of the findings presented here. Based on our predictions, the estimated annualized needs for anthelmintic drugs have been determined to be 10.2 million tablets. This amount should be further reviewed when the security issue in north-eastern Nigeria is resolved and prevalence data for this region become available to update model-based estimates. The fact that infection prevalence of STHs are considerably lower when compared to past estimates and projections [11] points to progress made, thanks to efforts by various governmental and non-governmental health development agencies implementing deworming programs across the country. Hence, these efforts should be sustained with adequate funding [12].

We fitted Bayesian geostatistical models to identify environmental and socioeconomic predictors that influence the distribution of each of the three STH species. Our results show that NDVI is a major environmental predictor for hookworm infection, while day LST is negatively associated with the distribution of *A*. *lumbricoides*. The results of our predictions are supported by the biology, ecology, and epidemiology of STHs. In fact, low humidity, associated with high temperature, leads to cessation of embryonation of *A*. *lumbricoides*, while high humidity promotes quick embryonation of *A*. *lumbricoides* eggs [48,49]. Our results are in line with earlier reports on the influence of temperature in the distribution and transmission of STH infections in Bolivia and the People’s Republic of China [37,50]. The observed prevalence data show that hookworm infection is the most widespread of the three common STH infections and has a higher predicted prevalence than the other two species. This finding is setting-dependent since other studies carried out in Bolivia, the People’s Republic of China, and Kenya found that *A*. *lumbricoides* is the predominant STH species [37,50,51].

The only socioeconomic predictor used (i.e., urban-rural classification) did not show any relationship with *A*. *lumbricoides* and *T*. *trichiura* infections. Other socioeconomic proxies, such as sanitation level and access to clean water, may be able to better explain the spatial distribution of infection risk with STHs [52]. However, unless individual information on both infection and, for instance, sanitation become available, such socioeconomic proxies might not improve predictions [29]. The predictors identified indicate that high night LST, which is often observed in the desert part of Nigeria as well as high altitude, can prohibit the survival of *T*. *trichiura*. This result may explain the quasi-absence of this STH species in the northern part of Nigeria (where there is extreme heat and a short wet season).

The strength of this study is that our analysis is based on recent survey data obtained by the FMoH Nigeria in 2011, adhering to standard and uniform diagnostic methods, focussing on school-aged children across all surveyed locations. This helps to avoid prediction bias associated with heterogeneities (e.g., due to diagnostic error and different age groups across surveys) arising from historically compiled data [52]. More importantly, an accurate and up-to-date map of STH infections is more reliable in making decisions for helminthiasis control, as relying on historic data alone may not give a true picture of the current status of the disease [53]. A limitation of this analysis is the lack of data from most of the north-eastern part of the country due to security issues and therefore prediction uncertainties are high in that part of the country.

There are two important points to consider in the calculation of treatments as well as number of people infected that are offered for discussion. First, the geographic level (such as district, state, and country) of aggregating pixel-level predictions has an impact on the overall result, as discussed by Schur *et al*. [34]. The treatments over an administrative area can be calculated using either pixel-level prevalence estimates (aggregated over the area) or population-adjusted prevalence over the area. In the present study we compared treatment needs calculated from both approaches using LGA as the level of aggregation. The total number of treatments for the whole country differs by 1,197,180 tablets between the two approaches. The main reason of this difference is that population-adjustment over an area (e.g., LGA) might be dominated by largely populated pixels that usually correspond to urban settlements. Second, the pixel resolution affects the number of estimated treatments. Low resolution would lead to few pixels covering large surfaces and crossing boundaries of administrative levels. Therefore, calculations do not take into account the variation in the population density and can wrongly assign treatments to areas.

Our estimates are lower than those recently reported in a geostatistical analysis of STH infection across sub-Saharan Africa [28]. The previous analysis used historic data over the past 50 years stemming from 33 states in Nigeria, some of which had high prevalence before 2000. It also considered a common temporal trend across all countries and showed a prevalence decrease after 2000, probably due to socioeconomic development as well as preventive chemotherapy that have been scaled up recently. In Nigeria, according to the preventive chemotherapy database of WHO, from 2003 to 2010, there have been more than 10 million school-aged children and almost 7 million preschool-aged children treated for STHs. From 2010 onwards, more than 22 million school-aged children have received preventive chemotherapy. In our study, using recent survey data, we predicted lower prevalence, indicating a continuous decline in the prevalence of STH.

In conclusion, we have produced spatially explicit model-based risk estimates of the geographic distribution of the three main species of STHs in Nigeria and determined underlying environmental risk factors. This is useful for planning the control of STH. We have further estimated the number school-aged children infected and at risk of infection, and provided annualized deworming requirement for Nigeria. With these data, the national STH control program can mobilize resources and attract local, national, and international support to escalate the implementation of preventive chemotherapy and other control measures nationwide.

## Supporting Information

S1 Fig The effect of land surface temperature (LST) at night. (TIF)

S2 Fig Maps of environmental co-variates used in the model. (DOCX)

S1 Table Population-adjusted and Pixel treatment requirement per local government areas (LGAs) in Nigeria. (DOCX)

S1 Text Details of analysis conducted. (DOCX)
