# Supplementary figures and images for: Bayesian Geostatistical Model-Based Estimates of Soil-Transmitted Helminth Infection in Nigeria, Including Annual Deworming Requirements
Source: PLoS Negl Trop Dis. 2015 Apr 24;9(4):e0003740. doi: 10.1371/journal.pntd.0003740 (PMC4409219; doi:10.1371/journal.pntd.0003740)

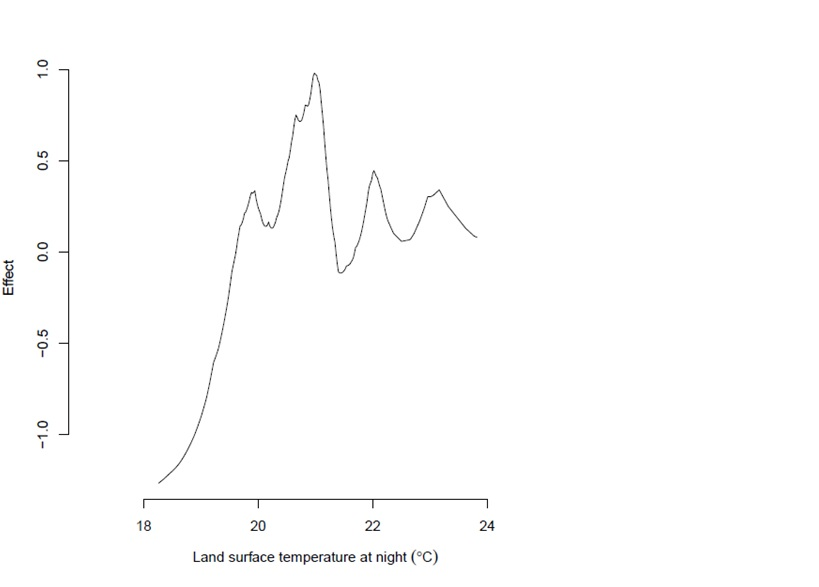

Supplement: S1 Fig — (TIF) [file pntd.0003740.s001.tif]
