# Supplementary material for: Bayesian Geostatistical Model-Based Estimates of Soil-Transmitted Helminth Infection in Nigeria, Including Annual Deworming Requirements
Source: PLoS Negl Trop Dis. 2015 Apr 24;9(4):e0003740. doi: 10.1371/journal.pntd.0003740 (PMC4409219; doi:10.1371/journal.pntd.0003740)
Supplement: S2 Fig — (DOCX) [file pntd.0003740.s002.docx]

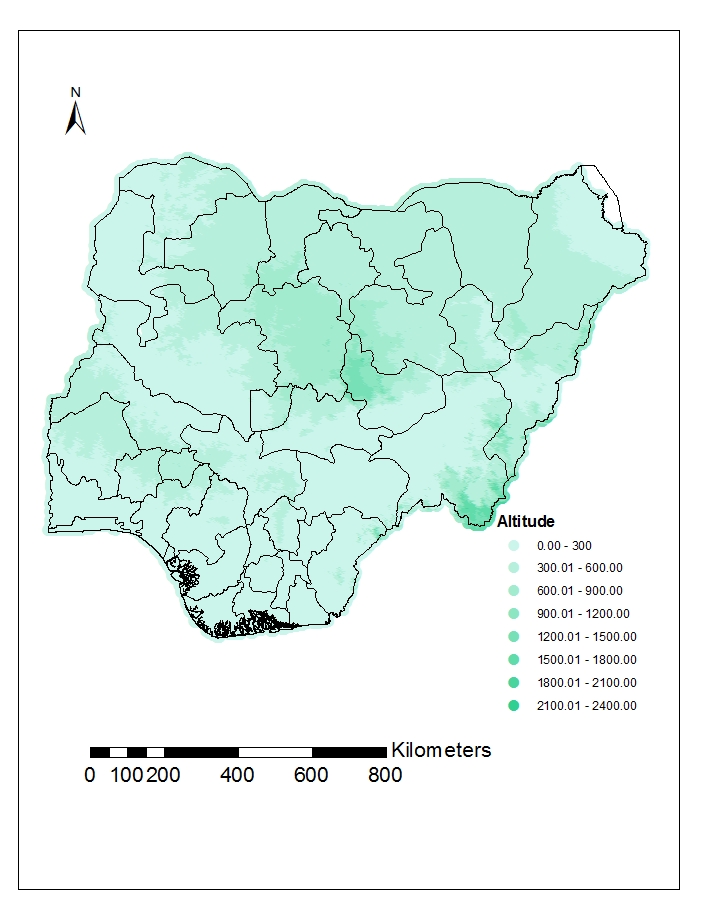


S2A Fig: Map of altitude


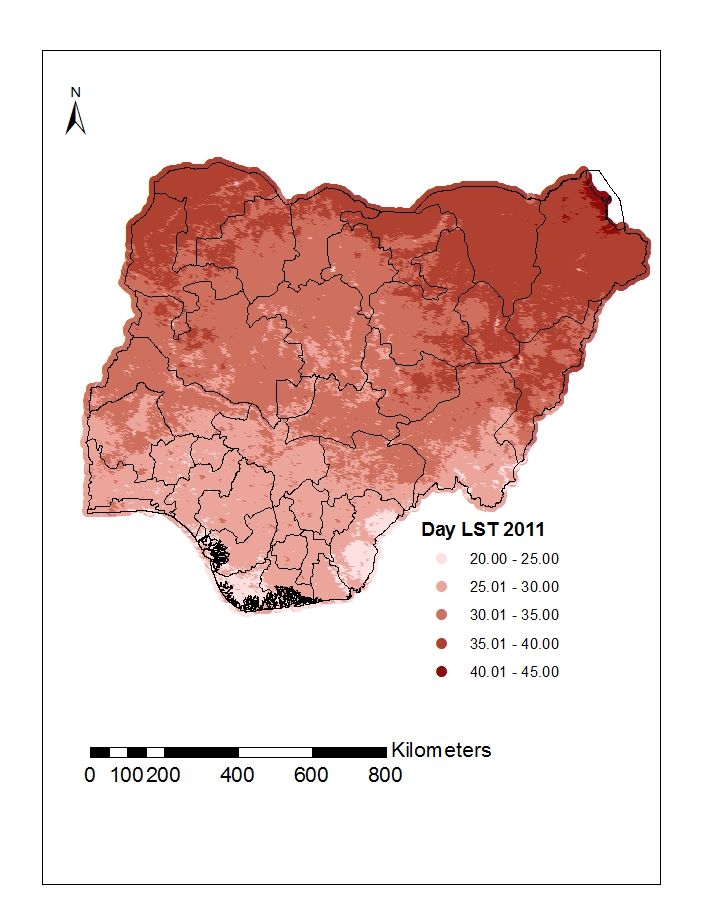


S2B Fig: Map of day Land surface temperature in 2011


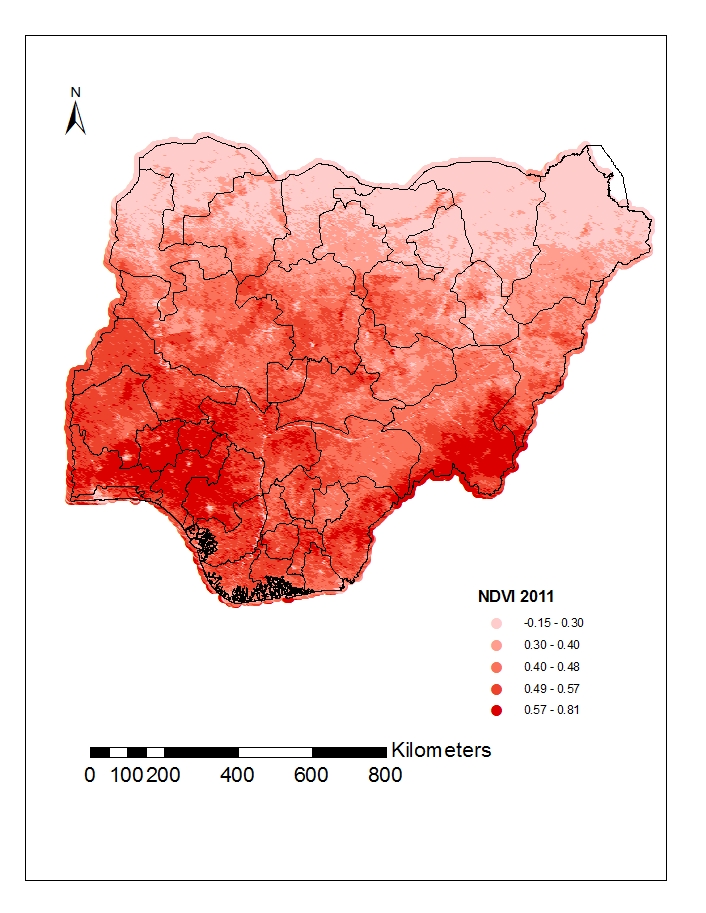


S2C Fig: Map of Normalize Differential Vegetation Index in 2011


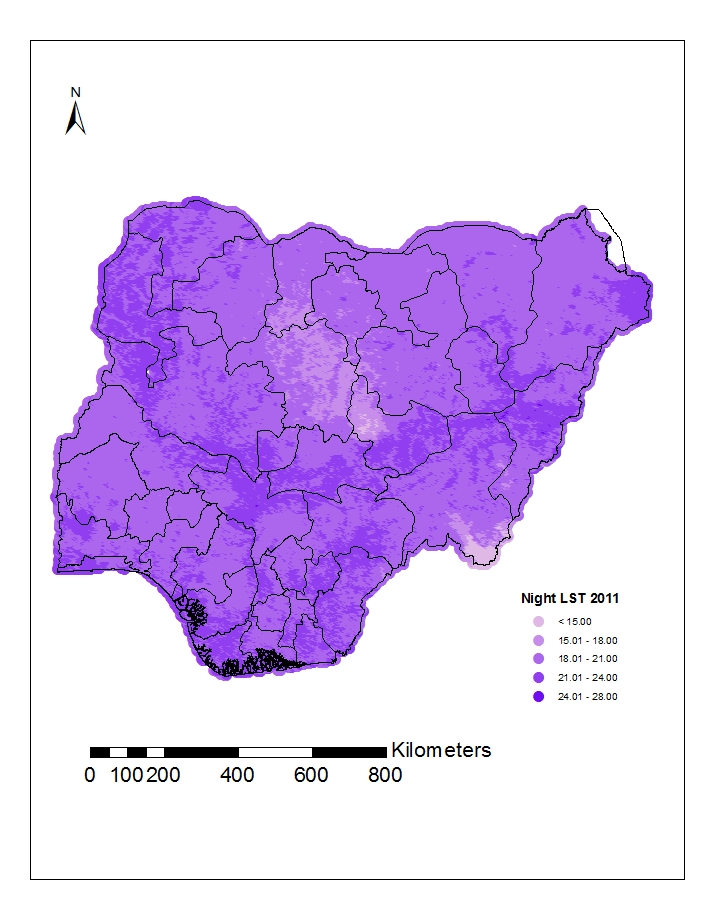


S2D Fig: Map of Night Land Surface Temperature in 2011


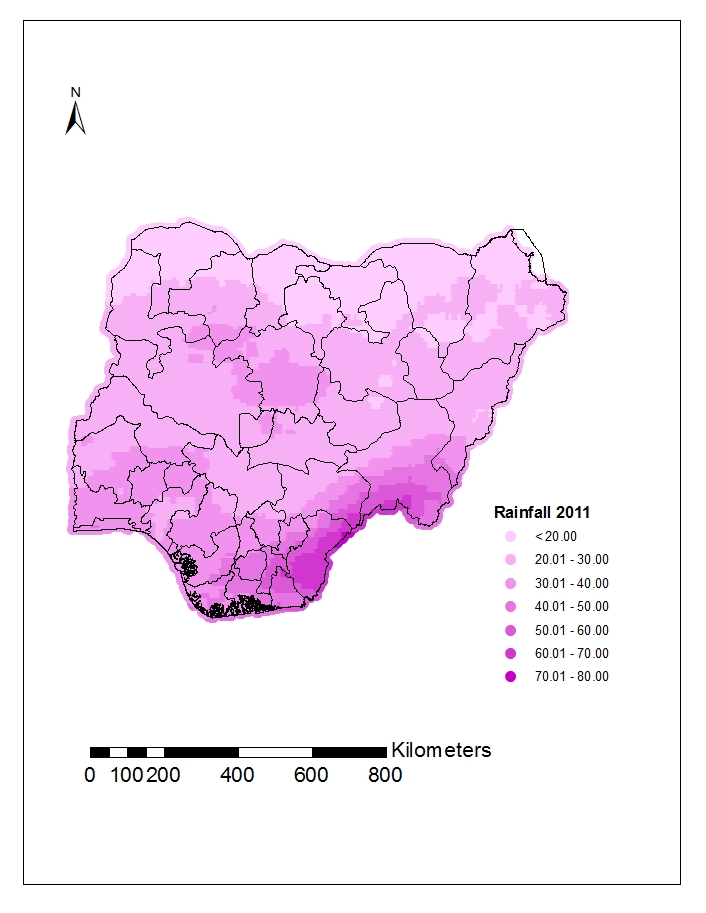


S2E Fig: Map of Rainfall in 2011


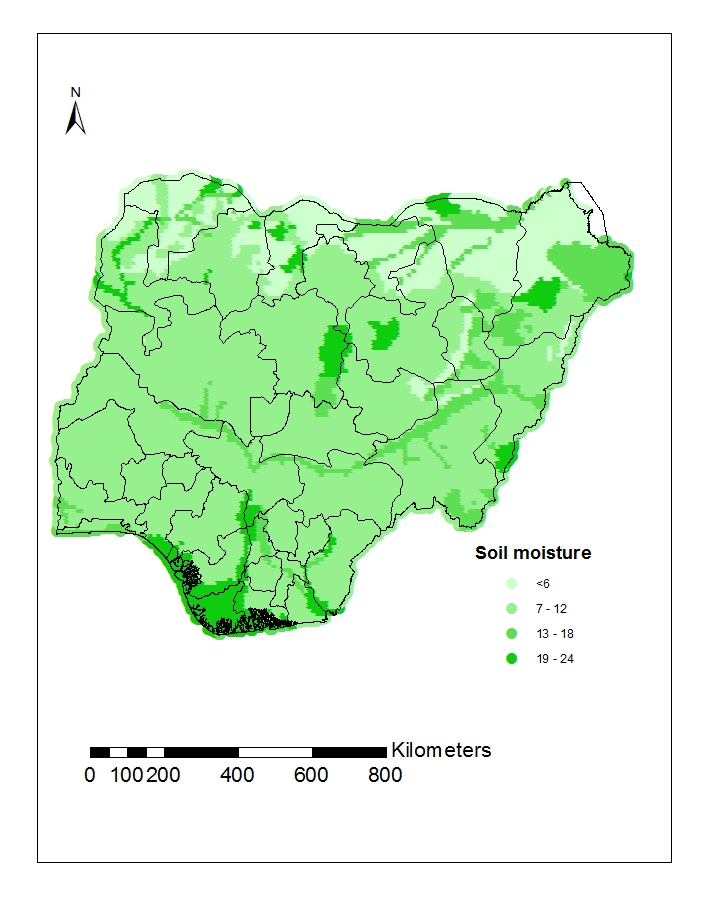


S2F Fig: Map of Soil moisture in 2011


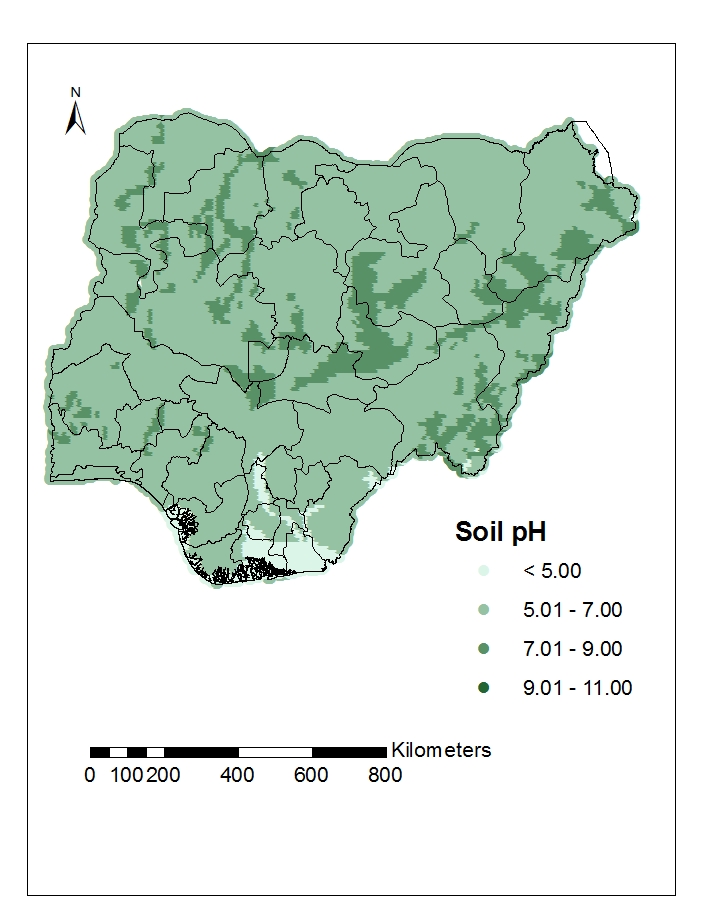


S2G Fig: Map of Soil pH in 2011
