# Supplementary material for: Bayesian Geostatistical Model-Based Estimates of Soil-Transmitted Helminth Infection in Nigeria, Including Annual Deworming Requirements
Source: PLoS Negl Trop Dis. 2015 Apr 24;9(4):e0003740. doi: 10.1371/journal.pntd.0003740 (PMC4409219; doi:10.1371/journal.pntd.0003740)
Supplement: S1 Table — (DOCX) [file pntd.0003740.s003.docx]

S1 Table 1: Treatment estimates according to the WHO cut offs based on two approaches: (i) using LGA-population-adjusted prevalence, and (ii) using pixel-based treatments and aggregated at LGA

| LGA | population -adjusted-based treatments | pixel-based treatments |
| --- | --- | --- |
| Aba North | 0 (0, 55693) | 79 (0, 55693) |
| Aba South | 0 (0, 96308) | 3005 (0, 96649) |
| Abadam | 0 (0, 0) | 129 (0, 7954) |
| Abaji | 0 (0, 14680) | 1824 (0, 7016) |
| Abak | 0 (0, 36011) | 7010 (0, 45624) |
| Abakalik | 0 (0, 0) | 1484 (0, 10054) |
| Abeokuta South | 0 (0, 77048) | 458 (0, 106510) |
| AbeokutaNorth | 0 (0, 43365) | 13554 (607, 40245) |
| Abi | 0 (0, 55643) | 17932 (0, 72542) |
| Aboh-Mba | 0 (0, 50047) | 17465 (0, 69047) |
| Abua-Odu | 0 (0, 76746) | 31379 (0, 99142) |
| AbujaMun | 0 (0, 0) | 10001 (207, 34338) |
| Adavi | 0 (0, 110768) | 7841 (0, 128115) |
| Ado | 0 (0, 47559) | 18100 (213, 48497) |
| Ado-Ekiti | 0 (0, 168630) | 2514 (0, 161243) |
| AdoOdo-Ota | 0 (0, 135484) | 48974 (5458, 114443) |
| Afijio | 0 (0, 39169) | 10193 (0, 42126) |
| Afikpo | 0 (0, 35784) | 3230 (0, 41559) |
| AfikpoSo | 0 (0, 42255) | 13977 (0, 56100) |
| Agaie | 0 (0, 38042) | 3347 (0, 17724) |
| Agatu | 0 (0, 37564) | 11363 (0, 43002) |
| Agege | 0 (0, 124610) | 0 (0, 116154) |
| Aguata | 0 (0, 28513) | 0 (0, 44962) |
| Agwara | 0 (0, 7062) | 882 (0, 11599) |
| Ahizu-Mb | 0 (0, 46682) | 11515 (0, 65565) |
| Ahoada East | 45090 (0, 45090) | 18465 (0, 61879) |
| Ahoada West | 0 (0, 64040) | 24203 (0, 84025) |
| Ajaokuta | 0 (0, 41961) | 11518 (59, 39469) |
| Ajeromi-Ifelodun | 0 (0, 105251) | 0 (0, 108445) |
| Ajingi | 0 (0, 86539) | 2000 (0, 63540) |
| Akamkpa | 0 (0, 65837) | 27567 (8072, 62370) |
| Akinyele | 0 (0, 58493) | 4093 (0, 54807) |
| Akko | 0 (0, 187950) | 6549 (0, 112461) |
| Akoko North-East | 0 (0, 58445) | 16642 (89, 76634) |
| Akoko South-East | 25242 (0, 50485) | 14382 (0, 41793) |
| Akoko South-West | 60524 (0, 60524) | 33061 (91, 86722) |
| Akoko-Ed | 73371 (0, 73371) | 34582 (1999, 86268) |
| AkokoNorthWest | 60349 (0, 120698) | 35751 (176, 89931) |
| Akpabuyo | 0 (0, 96224) | 23473 (0, 89700) |
| Akukutor | 0 (0, 14091) | 2560 (0, 10724) |
| Akure North | 59287 (59287, 59287) | 47110 (29316, 64460) |
| Akure South | 0 (0, 96238) | 22237 (2842, 81433) |
| Akwanga | 0 (0, 27993) | 3253 (2, 33051) |
| Albasu | 0 (0, 50935) | 999 (0, 31981) |
| Aleiro | 0 (0, 0) | 0 (0, 3659) |
| Alimosho | 0 (0, 313828) | 39463 (0, 326769) |
| Alkaleri | 0 (0, 0) | 9413 (596, 44253) |
| Amuwo Odofin | 0 (0, 192227) | 18376 (0, 266336) |
| Anambra East | 0 (0, 111928) | 25243 (1233, 90014) |
| Anambra West | 0 (0, 38337) | 8576 (193, 22387) |
| Anaocha | 0 (0, 0) | 0 (0, 0) |
| Andoni-O | 0 (0, 46897) | 7407 (0, 51602) |
| Aninri | 0 (0, 24517) | 3424 (0, 28172) |
| AniochaN | 0 (0, 33212) | 10982 (0, 35972) |
| AniochaS | 40369 (0, 51470) | 17624 (159, 60665) |
| Anka | 0 (0, 0) | 2326 (0, 14441) |
| Ankpa | 0 (0, 81507) | 25606 (870, 93963) |
| Apa | 0 (0, 26311) | 5971 (0, 28048) |
| Apapa | 0 (0, 210193) | 0 (0, 221049) |
| Ardo-Kola | 59243 (42951, 59243) | 49623 (19962, 58225) |
| Arewa | 0 (0, 0) | 1704 (0, 27794) |
| Argungu | 0 (0, 59268) | 391 (0, 33425) |
| Arochukw | 0 (0, 44415) | 14430 (1, 54079) |
| Asa | 233141 (116570, 233141) | 190846 (145222, 203662) |
| Asari-To | 0 (0, 80284) | 5127 (0, 63109) |
| Askira-U | 0 (0, 54956) | 2675 (0, 32111) |
| Atakumosa East | 0 (0, 90512) | 4623 (569, 64340) |
| Atakumosa West | 0 (0, 25154) | 5392 (0, 22158) |
| Atiba | 0 (0, 43009) | 12557 (2509, 40486) |
| Atisbo | 0 (0, 27485) | 11367 (1714, 24229) |
| Augie | 0 (0, 0) | 0 (0, 12409) |
| Auyo | 0 (0, 0) | 7 (0, 4576) |
| Awe | 0 (0, 32825) | 6319 (249, 25392) |
| Awgu | 0 (0, 62264) | 7934 (0, 60243) |
| AwkaNort | 41900 (0, 41900) | 20961 (5626, 33872) |
| AwkaSout | 0 (0, 0) | 501 (0, 13597) |
| Ayamelum | 0 (0, 46767) | 14763 (3575, 28198) |
| Ayedaade | 0 (0, 50268) | 1667 (59, 52761) |
| Ayedire | 0 (0, 12097) | 1138 (0, 14844) |
| Babura | 0 (0, 48749) | 1035 (0, 24791) |
| Badagary | 0 (0, 76876) | 28247 (3248, 75107) |
| Bade | 0 (0, 0) | 130 (0, 10441) |
| Bagudo | 0 (0, 74590) | 5277 (236, 39329) |
| Bagwai | 0 (0, 37086) | 3734 (0, 34693) |
| Bakassi | 0 (0, 3599) | 160 (0, 2078) |
| Bakori | 0 (0, 42587) | 4898 (0, 34022) |
| Bakura | 0 (0, 0) | 252 (0, 5072) |
| Balanga | 0 (0, 75563) | 4385 (0, 40618) |
| Bali | 57050 (0, 57050) | 27505 (13089, 52341) |
| Bama | 0 (0, 0) | 2388 (0, 23477) |
| Barkin Ladi | 0 (0, 46656) | 9785 (0, 44857) |
| Baruten | 63840 (0, 63840) | 31845 (20311, 48783) |
| Bassa | 0 (0, 44157) | 14769 (2470, 40390) |
| Bassa | 0 (0, 46642) | 12940 (314, 37581) |
| Batagarawa | 0 (0, 51939) | 49 (0, 33853) |
| Batsari | 0 (0, 55921) | 1275 (0, 41443) |
| Bauchi | 0 (0, 167755) | 6418 (375, 105163) |
| Baure | 0 (0, 0) | 46 (0, 27235) |
| Bayo | 0 (0, 22235) | 151 (0, 12443) |
| Bebeji | 0 (0, 63645) | 7522 (0, 57904) |
| Bekwarra | 0 (0, 37142) | 10296 (0, 43882) |
| Bende | 0 (0, 58181) | 16882 (0, 73643) |
| Biase | 0 (0, 47517) | 20186 (1834, 56877) |
| Bichi | 0 (0, 96263) | 6027 (0, 76162) |
| Bida | 0 (0, 54827) | 0 (0, 27012) |
| Billiri | 0 (0, 56066) | 1970 (0, 34761) |
| Bindawa | 0 (0, 40435) | 0 (0, 27510) |
| Binji | 0 (0, 0) | 0 (0, 45) |
| Biriniwa | 0 (0, 0) | 0 (0, 6955) |
| Birnin-G | 0 (0, 80085) | 22788 (5351, 58572) |
| BirninKe | 0 (0, 69870) | 329 (0, 56600) |
| BirninKu | 0 (0, 0) | 1174 (0, 29366) |
| Birnin-Magaji-Kiyaw | 0 (0, 45496) | 6737 (593, 27665) |
| Biu | 0 (0, 48659) | 1164 (0, 33837) |
| Bodinga | 0 (0, 0) | 0 (0, 8343) |
| Bogoro | 0 (0, 17776) | 992 (0, 15247) |
| Boki | 55495 (0, 55495) | 23992 (3104, 59836) |
| Bokkos | 0 (0, 46503) | 13540 (440, 43442) |
| Boluwaduro | 0 (0, 34055) | 2617 (0, 26892) |
| Bomadi | 0 (0, 21090) | 8544 (0, 25677) |
| Bonny | 0 (0, 19990) | 5276 (0, 44829) |
| Borgu | 0 (0, 0) | 8265 (1497, 22020) |
| Boripe | 0 (0, 19487) | 3289 (0, 21383) |
| Borsari | 0 (0, 0) | 1143 (0, 14570) |
| Bosso | 0 (0, 61524) | 7091 (0, 41604) |
| Brass | 0 (0, 0) | 11884 (317, 31182) |
| Buji | 0 (0, 7585) | 98 (0, 12346) |
| Bukkuyum | 0 (0, 0) | 3106 (15, 24469) |
| Bungudu | 0 (0, 0) | 3049 (0, 14966) |
| Bunkure | 0 (0, 50955) | 5475 (0, 48005) |
| Bunza | 0 (0, 31563) | 0 (0, 24551) |
| Buruku | 0 (0, 65541) | 18618 (11112, 32588) |
| Burutu | 0 (0, 56624) | 23180 (2202, 60238) |
| Bwari | 0 (0, 82269) | 13274 (31, 49892) |
| Calabar | 0 (0, 94198) | 6714 (0, 87718) |
| Calabar South | 0 (0, 0) | 16 (0, 1539) |
| Chanchaga | 0 (0, 0) | 0 (0, 2819) |
| Charanchi | 0 (0, 36436) | 974 (0, 36574) |
| Chibok | 0 (0, 19132) | 428 (0, 12998) |
| Chikun | 0 (0, 111132) | 31883 (6834, 80791) |
| Dala | 0 (0, 24203) | 0 (0, 24203) |
| Damaturu | 0 (0, 8346) | 294 (0, 19305) |
| Damban | 0 (0, 38215) | 55 (0, 29751) |
| Dambatta | 0 (0, 74015) | 1143 (0, 39736) |
| Damboa | 0 (0, 0) | 4103 (0, 34727) |
| Dandi | 0 (0, 51588) | 1539 (0, 29386) |
| Dandume | 0 (0, 39722) | 4871 (0, 39046) |
| Dange-Shuni | 0 (0, 0) | 147 (0, 4528) |
| Danja | 0 (0, 33259) | 5118 (0, 28883) |
| Danko Wasagu | 0 (0, 66338) | 8710 (461, 34062) |
| Danmusa | 0 (0, 33388) | 3662 (0, 30914) |
| Darazo | 0 (0, 0) | 1829 (0, 18212) |
| Dass | 0 (0, 22984) | 4029 (0, 20303) |
| Daura | 0 (0, 61813) | 0 (0, 40165) |
| DawakinK | 0 (0, 53179) | 6825 (0, 47579) |
| DawakinT | 0 (0, 73224) | 5036 (0, 58618) |
| Degema | 0 (0, 84671) | 13141 (0, 66878) |
| Dekina | 73756 (0, 73756) | 30807 (3869, 85570) |
| Demsa | 0 (0, 0) | 2405 (0, 43975) |
| Dikwa | 0 (0, 27967) | 397 (0, 16290) |
| Doguwa | 0 (0, 47372) | 11214 (966, 43765) |
| Doma | 0 (0, 37638) | 7009 (597, 27217) |
| Donga | 37398 (0, 37398) | 18639 (7621, 37747) |
| Dukku | 0 (0, 59478) | 4867 (250, 30681) |
| Dunukofia | 0 (0, 38948) | 202 (0, 19650) |
| Dutse | 0 (0, 77233) | 2189 (0, 59128) |
| Dutsi | 0 (0, 33095) | 0 (0, 30166) |
| Dutsin-M | 0 (0, 55239) | 1951 (0, 57751) |
| Eastern Obolo | 0 (0, 13525) | 3021 (0, 12733) |
| Ebonyi | 0 (0, 57725) | 2724 (0, 33575) |
| Edati | 0 (0, 10782) | 469 (0, 7111) |
| Ede North | 0 (0, 12757) | 285 (0, 14243) |
| Ede South | 0 (0, 14177) | 1960 (0, 18216) |
| Edu | 0 (0, 57036) | 4726 (115, 23107) |
| Efon | 0 (0, 36288) | 2765 (0, 46590) |
| EgbadoNorth | 0 (0, 51234) | 12797 (1156, 42706) |
| EgbadoSouth | 0 (0, 48314) | 11079 (5, 44777) |
| Egbeda | 0 (0, 98486) | 2005 (0, 86693) |
| Egbedore | 0 (0, 49692) | 2861 (0, 58112) |
| Egor | 0 (0, 83487) | 2399 (0, 89016) |
| Ehime-Mb | 0 (0, 39586) | 8950 (0, 51871) |
| Ejigbo | 0 (0, 41594) | 10689 (0, 51812) |
| Ekeremor | 0 (0, 76073) | 25575 (2205, 74462) |
| Eket | 0 (0, 67093) | 5755 (0, 77875) |
| Ekiti | 17815 (0, 17815) | 10970 (5682, 16684) |
| EkitiEas | 20567 (0, 41133) | 15619 (0, 35525) |
| EkitiSouth-West | 0 (0, 37017) | 141 (0, 23128) |
| EkitiWest | 0 (0, 38869) | 5975 (0, 44057) |
| Ekwusigo | 0 (0, 0) | 0 (0, 3428) |
| Eleme | 0 (0, 62277) | 14312 (0, 72569) |
| Emuoha | 61567 (0, 61567) | 25785 (855, 74326) |
| Emure-Ise-Orun | 51159 (0, 102318) | 43021 (68, 89845) |
| Enugu East | 0 (0, 49373) | 8620 (0, 33503) |
| Enugu North | 0 (0, 125906) | 14724 (0, 124089) |
| EnuguSou | 0 (0, 55651) | 3198 (0, 64014) |
| Epe | 0 (0, 56451) | 2213 (5, 32957) |
| EsanCent | 27297 (0, 34804) | 11769 (0, 38984) |
| EsanNort | 0 (0, 71887) | 11714 (0, 59257) |
| EsanSout | 0 (0, 50241) | 19678 (197, 59066) |
| EsanWest | 0 (0, 38731) | 6855 (0, 44663) |
| Ese-Odo | 41019 (41019, 82038) | 41537 (20378, 66815) |
| Esit Eket | 0 (0, 16218) | 2094 (0, 21160) |
| Essien-U | 0 (0, 102243) | 11181 (0, 79318) |
| Etche | 23649 (0, 94594) | 19858 (0, 70196) |
| Ethiope West | 0 (0, 90012) | 14656 (0, 107539) |
| EthiopeE | 0 (0, 55831) | 19117 (0, 75963) |
| EtimEkpo | 0 (0, 25469) | 6046 (0, 33501) |
| Etinan | 0 (0, 51597) | 17980 (0, 66984) |
| Eti-Osa | 0 (0, 0) | 0 (0, 43016) |
| Etsako Central | 0 (0, 25925) | 9988 (0, 33977) |
| EtsakoEa | 0 (0, 46058) | 18535 (484, 56796) |
| EtsakoWe | 0 (0, 64531) | 17064 (142, 58502) |
| Etung | 0 (0, 22689) | 8579 (79, 27656) |
| Ewekoro | 0 (0, 16635) | 3250 (0, 14837) |
| Ezeagu | 0 (0, 0) | 2001 (0, 15252) |
| Ezinihit | 0 (0, 77438) | 11620 (0, 69849) |
| Ezza North | 0 (0, 38015) | 3401 (0, 40538) |
| Ezza South | 0 (0, 39874) | 7588 (0, 54213) |
| Fagge | 0 (0, 193797) | 0 (0, 193797) |
| Fakai | 0 (0, 31639) | 2031 (0, 20724) |
| Faskari | 0 (0, 57103) | 8743 (249, 28681) |
| Fika | 0 (0, 0) | 780 (0, 14619) |
| Fufore | 0 (0, 63367) | 8812 (656, 28778) |
| Funakaye | 0 (0, 75786) | 865 (0, 57599) |
| Fune | 0 (0, 0) | 2989 (0, 33704) |
| Funtua | 0 (0, 73797) | 3312 (0, 71663) |
| Gabasawa | 0 (0, 66796) | 1400 (0, 44278) |
| Gada | 0 (0, 0) | 0 (0, 1889) |
| Gagarawa | 0 (0, 0) | 0 (0, 3740) |
| Gamawa | 0 (0, 81679) | 3254 (0, 59183) |
| Gamjuwa | 0 (0, 0) | 6405 (705, 29590) |
| Ganye | 0 (0, 49662) | 16799 (1194, 50346) |
| Garki | 0 (0, 11096) | 1586 (0, 19586) |
| Garko | 0 (0, 46422) | 4926 (0, 36237) |
| Garum Mallam | 0 (0, 29956) | 2244 (0, 33807) |
| Gashaka | 24298 (0, 24298) | 14260 (5330, 23917) |
| Gassol | 0 (0, 72908) | 15904 (3371, 48064) |
| Gaya | 0 (0, 34712) | 1322 (0, 25822) |
| Gbako | 0 (0, 39025) | 4999 (0, 26307) |
| Gboko | 114558 (0, 114558) | 42972 (15880, 109378) |
| Gboyin | 29115 (0, 58230) | 15393 (51, 44278) |
| Geidam | 0 (0, 45416) | 2290 (0, 23221) |
| Gezawa | 0 (0, 151434) | 7193 (0, 153874) |
| Giade | 0 (0, 34987) | 297 (0, 18029) |
| Girie | 0 (0, 34022) | 1942 (0, 21706) |
| Giwa | 0 (0, 80890) | 16643 (787, 64479) |
| Gokana | 0 (0, 57327) | 3933 (0, 85565) |
| Gombe | 0 (0, 420) | 0 (0, 420) |
| Gombi | 0 (0, 28608) | 690 (0, 13099) |
| Goronyo | 0 (0, 50372) | 6950 (8, 30305) |
| Gubio | 0 (0, 0) | 1118 (0, 20972) |
| Gudu | 0 (0, 0) | 38 (0, 6227) |
| Gujba | 0 (0, 37455) | 1380 (0, 18589) |
| Gulani | 0 (0, 0) | 480 (0, 12236) |
| Guma | 0 (0, 59580) | 17777 (2776, 53423) |
| Gumel | 0 (0, 0) | 0 (0, 8410) |
| Gummi | 0 (0, 0) | 2652 (15, 18809) |
| Gurara | 0 (0, 36360) | 6131 (80, 29543) |
| Guri | 0 (0, 0) | 471 (0, 11509) |
| Gusau | 0 (0, 0) | 17254 (3605, 40927) |
| Guyuk | 0 (0, 33309) | 639 (0, 17074) |
| Guzamala | 0 (0, 28757) | 291 (0, 14348) |
| Gwadabaw | 0 (0, 0) | 1530 (0, 18783) |
| Gwagwala | 0 (0, 0) | 1069 (0, 10011) |
| Gwale | 0 (0, 98499) | 0 (0, 116567) |
| Gwandu | 0 (0, 61100) | 15421 (0, 50882) |
| Gwaram | 0 (0, 0) | 3200 (0, 23667) |
| Gwarzo | 0 (0, 49780) | 6083 (0, 51612) |
| Gwer East | 61501 (0, 61501) | 31265 (4868, 68375) |
| GwerWest | 0 (0, 35842) | 8697 (128, 36788) |
| Gwiwa | 0 (0, 43174) | 108 (0, 22365) |
| Gwoza | 0 (0, 64971) | 2749 (0, 34215) |
| Hadejia | 0 (0, 0) | 0 (0, 4782) |
| Hawul | 0 (0, 37783) | 2105 (0, 22518) |
| Hong | 0 (0, 74702) | 3311 (0, 38283) |
| IbadanNorth | 0 (0, 79567) | 0 (0, 78741) |
| IbadanNorth-East | 0 (0, 23007) | 0 (0, 35911) |
| IbadanNorth-West | 0 (0, 42629) | 0 (0, 42008) |
| IbadanSouth-East | 0 (0, 103164) | 0 (0, 98423) |
| IbadanSouth-West | 0 (0, 83629) | 0 (0, 76619) |
| Ibaji | 0 (0, 36392) | 15495 (974, 35909) |
| Ibarapa Central | 0 (0, 43652) | 1244 (0, 48973) |
| Ibarapa East | 0 (0, 32627) | 4626 (0, 27183) |
| Ibarapa North | 0 (0, 28704) | 3751 (25, 25534) |
| Ibeju-Lekki | 0 (0, 0) | 1875 (0, 17952) |
| Ibeno | 0 (0, 18284) | 3300 (0, 14799) |
| Ibesikpo Asutan | 0 (0, 42410) | 13961 (0, 58212) |
| Ibi | 0 (0, 25376) | 6049 (1117, 16047) |
| Ibiono Ibom | 0 (0, 45152) | 16948 (0, 63968) |
| Idah | 0 (0, 11763) | 277 (0, 17116) |
| Idanre | 38375 (0, 38375) | 18020 (4522, 40667) |
| Ideato South | 0 (0, 34615) | 4559 (0, 40707) |
| IdeatoNo | 0 (0, 48547) | 5710 (0, 46326) |
| Idemili North | 0 (0, 0) | 0 (0, 0) |
| Idemili South | 0 (0, 0) | 0 (0, 156) |
| Ido | 0 (0, 29832) | 6261 (93, 24188) |
| Ido-Osi | 0 (0, 47256) | 10663 (0, 65579) |
| Ifako-Ijaye | 0 (0, 113082) | 0 (0, 124551) |
| Ife East | 0 (0, 13689) | 210 (0, 13031) |
| Ife North | 0 (0, 130512) | 1794 (0, 123204) |
| Ife South | 0 (0, 9804) | 819 (0, 16762) |
| IfeCentral | 0 (0, 12096) | 1 (0, 13152) |
| Ifedayo | 0 (0, 9659) | 1483 (0, 11457) |
| Ifedore | 0 (0, 0) | 154 (0, 969) |
| Ifelodun | 0 (0, 61276) | 17698 (9851, 30272) |
| Ifelodun | 0 (0, 54508) | 3716 (0, 63840) |
| Ifo | 0 (0, 136908) | 3751 (0, 87511) |
| Igabi | 0 (0, 199781) | 33852 (1241, 142541) |
| Igalamela-Odolu | 0 (0, 56341) | 19427 (980, 57723) |
| Igbo-Eti | 0 (0, 62425) | 1721 (0, 40953) |
| Igbo-eze North | 0 (0, 94242) | 21597 (0, 110842) |
| Igbo-eze South | 0 (0, 37165) | 4933 (0, 31128) |
| Igueben | 17536 (0, 35073) | 8214 (0, 26764) |
| Ihiala | 0 (0, 60377) | 17116 (2050, 49073) |
| Ihitte-U | 0 (0, 40689) | 7042 (0, 42120) |
| Ijebu North-East | 17512 (0, 35025) | 8728 (70, 25896) |
| IjebuEast | 38286 (0, 38286) | 19274 (5346, 36146) |
| IjebuNorth | 82460 (0, 164921) | 38138 (3547, 137059) |
| IjebuOde | 0 (0, 48755) | 5907 (0, 59260) |
| Ijero | 0 (0, 71124) | 4179 (0, 75707) |
| Ijumu | 23726 (0, 23726) | 12970 (659, 30763) |
| Ika | 0 (0, 24244) | 5097 (0, 32795) |
| IkaNorth | 0 (0, 51333) | 19383 (0, 59211) |
| Ikara | 0 (0, 54383) | 6015 (0, 42909) |
| IkaSouth | 0 (0, 53590) | 9359 (0, 60304) |
| Ikeduru | 0 (0, 47617) | 12865 (0, 59163) |
| Ikeja | 0 (0, 183888) | 0 (0, 129520) |
| Ikenne | 35014 (0, 70028) | 17844 (0, 60075) |
| Ikere | 0 (0, 44806) | 1356 (0, 44326) |
| Ikole | 47760 (0, 47760) | 20918 (37, 59039) |
| Ikom | 0 (0, 59144) | 14699 (1331, 62801) |
| Ikono | 0 (0, 37477) | 13285 (0, 48438) |
| Ikorodu | 0 (0, 162511) | 15771 (0, 161047) |
| Ikot-Aba | 0 (0, 38553) | 4893 (0, 44538) |
| Ikot-Ekp | 0 (0, 98006) | 11957 (0, 89265) |
| Ikpoba-Okha | 0 (0, 181373) | 17133 (129, 178939) |
| Ikwerre | 0 (0, 59064) | 21472 (0, 74779) |
| Ikwo | 0 (0, 50705) | 13750 (0, 55153) |
| Ikwuano | 0 (0, 68245) | 11181 (0, 51097) |
| Ila | 0 (0, 24712) | 4335 (0, 33868) |
| IlajeEseodo | 81602 (0, 81602) | 61711 (29075, 92498) |
| Ilejemeje | 14707 (0, 29413) | 6644 (0, 26336) |
| IleOluji-Okeigbo | 0 (0, 0) | 982 (0, 7622) |
| Ilesha East | 0 (0, 46004) | 1027 (0, 38330) |
| Ilesha West | 0 (0, 36671) | 399 (0, 49350) |
| Illela | 0 (0, 0) | 1299 (0, 28456) |
| Ilorin East | 0 (0, 56843) | 16291 (1404, 34739) |
| Ilorin South | 0 (0, 0) | 6579 (2992, 11887) |
| IlorinWe | 156816 (78408, 156816) | 115274 (90480, 142482) |
| Imeko-Afon | 0 (0, 19015) | 4033 (340, 13593) |
| Ingawa | 0 (0, 45109) | 658 (0, 27369) |
| Ini | 0 (0, 36298) | 13187 (0, 42661) |
| Ipokia | 0 (0, 32026) | 14036 (394, 34263) |
| Irele | 36021 (36021, 36021) | 35719 (21487, 51249) |
| Irepo | 0 (0, 36938) | 11684 (1033, 42934) |
| Irepodun | 38587 (0, 38587) | 25923 (12607, 42879) |
| Irepodun | 0 (0, 41960) | 1356 (0, 37467) |
| Irepodun-Ifelodun | 0 (0, 55899) | 11789 (0, 65889) |
| Irewole | 0 (0, 77775) | 1384 (0, 77654) |
| Isa | 0 (0, 0) | 153 (0, 12006) |
| Ise-Orun | 28868 (0, 57736) | 20301 (1339, 45986) |
| Iseyin | 0 (0, 75976) | 8982 (63, 65067) |
| Ishielu | 0 (0, 43660) | 9166 (15, 38474) |
| Isiala Ngwa North | 0 (0, 77466) | 14863 (0, 56146) |
| Isiala Ngwa South | 0 (0, 45608) | 14460 (0, 49235) |
| IsialaMb | 0 (0, 50878) | 19546 (0, 66123) |
| Isin | 22072 (0, 22072) | 17789 (9537, 29443) |
| Isi-Uzo | 0 (0, 45236) | 2170 (0, 25585) |
| Isokan | 0 (0, 3154) | 442 (0, 3482) |
| IsokoNor | 0 (0, 41206) | 15223 (0, 57054) |
| IsokoSou | 32268 (0, 64536) | 26795 (0, 80473) |
| Isu | 0 (0, 70271) | 3714 (0, 53391) |
| Isuikwua | 0 (0, 34359) | 6814 (0, 37373) |
| Itas-Gad | 0 (0, 0) | 1356 (0, 20844) |
| Itesiwaju | 0 (0, 34456) | 9602 (5, 35569) |
| Itu | 0 (0, 73743) | 18214 (0, 88359) |
| Ivo | 0 (0, 36966) | 11562 (0, 45992) |
| Iwajowa | 0 (0, 27452) | 9223 (576, 28493) |
| Iwo | 0 (0, 69741) | 4484 (0, 69740) |
| Izzi | 0 (0, 67132) | 14811 (134, 42524) |
| Jaba | 0 (0, 44492) | 6821 (0, 49669) |
| Jada | 0 (0, 25942) | 5361 (81, 24543) |
| Jahun | 0 (0, 0) | 836 (0, 18693) |
| Jakusko | 0 (0, 0) | 2628 (0, 22032) |
| Jalingo | 18061 (0, 18061) | 15160 (1850, 18061) |
| Jama'are | 0 (0, 40205) | 13 (0, 30130) |
| Jega | 0 (0, 0) | 0 (0, 10463) |
| Jema'a | 0 (0, 66680) | 18124 (610, 60845) |
| Jere | 0 (0, 26446) | 312 (0, 14746) |
| Jibia | 0 (0, 51404) | 1311 (0, 31318) |
| Jos East | 0 (0, 39827) | 7460 (213, 43525) |
| Jos North | 0 (0, 166069) | 18369 (0, 153699) |
| Jos South | 0 (0, 61436) | 8075 (0, 56628) |
| Kabba-Bu | 25319 (0, 50639) | 22102 (2916, 54171) |
| Kabo | 0 (0, 35746) | 2312 (0, 46856) |
| Kachia | 0 (0, 76043) | 23016 (5016, 51330) |
| Kaduna North | 0 (0, 79168) | 0 (0, 78479) |
| Kaduna South | 0 (0, 92129) | 0 (0, 92130) |
| KafinHau | 0 (0, 0) | 33 (0, 13424) |
| Kafur | 0 (0, 56910) | 9863 (0, 49320) |
| Kaga | 0 (0, 22580) | 929 (0, 13523) |
| Kagarko | 0 (0, 88388) | 25800 (2076, 66309) |
| Kaiama | 0 (0, 38118) | 11037 (3517, 25958) |
| Kaita | 0 (0, 54368) | 424 (0, 30589) |
| Kajola | 0 (0, 56297) | 8744 (0, 75518) |
| Kajuru | 0 (0, 54420) | 15466 (668, 49107) |
| Kala-Balge | 0 (0, 12530) | 22 (0, 7328) |
| Kalgo | 0 (0, 0) | 0 (0, 6461) |
| Kaltungo | 0 (0, 44659) | 2247 (0, 38116) |
| Kanam | 0 (0, 58221) | 4383 (0, 32440) |
| Kankara | 0 (0, 69010) | 13760 (3, 60359) |
| Kanke | 0 (0, 48668) | 5933 (0, 41123) |
| Kankiya | 0 (0, 52363) | 2252 (0, 39126) |
| Kano | 0 (0, 38367) | 0 (0, 41792) |
| Karasuwa | 0 (0, 40310) | 82 (0, 29972) |
| Karaye | 0 (0, 38229) | 5030 (0, 41346) |
| Karim-La | 0 (0, 68461) | 12630 (3082, 37033) |
| Karu | 0 (0, 82892) | 20186 (1909, 62058) |
| Katagum | 0 (0, 0) | 3456 (0, 59128) |
| Katcha | 0 (0, 31140) | 3885 (0, 19510) |
| Katsina (Benue) | 82127 (0, 82127) | 41351 (19491, 59347) |
| Katsina (K) | 0 (0, 106128) | 0 (0, 104200) |
| Kaugama | 0 (0, 0) | 4 (0, 2941) |
| Kaura | 0 (0, 53101) | 6959 (0, 62420) |
| Kaura-Na | 0 (0, 0) | 2645 (0, 15816) |
| Kauru | 0 (0, 31945) | 8804 (1508, 29804) |
| Kazaure | 0 (0, 52559) | 965 (0, 33265) |
| Keana | 0 (0, 23187) | 3540 (0, 24276) |
| Kebbe | 0 (0, 0) | 2198 (0, 13828) |
| Keffi | 0 (0, 27089) | 245 (0, 18006) |
| Khana | 0 (0, 84882) | 24744 (0, 104979) |
| Kibiya | 0 (0, 35290) | 1846 (0, 27719) |
| Kirfi | 0 (0, 44800) | 3827 (0, 24466) |
| KiriKasa | 0 (0, 0) | 67 (0, 12281) |
| Kiru | 0 (0, 64546) | 10679 (0, 50186) |
| Kiyawa | 0 (0, 53048) | 4449 (0, 29236) |
| Koko-Bes | 0 (0, 41074) | 551 (0, 20949) |
| Kokona | 0 (0, 32564) | 7696 (91, 28724) |
| Kolokuma-Opokuma | 0 (0, 28803) | 12048 (0, 34947) |
| Konduga | 0 (0, 184779) | 3790 (60, 118106) |
| Konshish | 0 (0, 59939) | 23463 (2104, 63233) |
| Kontogur | 0 (0, 48428) | 1551 (18, 42598) |
| Kosofe | 0 (0, 183549) | 596 (0, 182596) |
| Kotonkar | 0 (0, 32944) | 8566 (510, 36467) |
| Kubau | 0 (0, 76661) | 16628 (981, 60702) |
| Kudan | 0 (0, 35892) | 2685 (0, 35382) |
| Kuje | 0 (0, 26359) | 5390 (150, 16425) |
| Kukawa | 0 (0, 0) | 137 (0, 14413) |
| Kumbotso | 0 (0, 80087) | 1666 (0, 85641) |
| Kunchi | 0 (0, 30540) | 2065 (0, 21147) |
| Kura | 0 (0, 46617) | 3281 (0, 57535) |
| Kurfi | 0 (0, 33208) | 457 (0, 27411) |
| Kurmi | 29311 (0, 29311) | 20784 (9383, 32295) |
| Kusada | 0 (0, 32198) | 114 (0, 24292) |
| Kwali | 0 (0, 0) | 759 (0, 6450) |
| Kwami | 0 (0, 48738) | 2480 (0, 29322) |
| Kwande | 61448 (0, 61448) | 39545 (23005, 61276) |
| Kware | 0 (0, 0) | 0 (0, 2132) |
| Kwaya Kusar | 0 (0, 14310) | 282 (0, 9073) |
| Lafia | 0 (0, 103592) | 7919 (694, 80749) |
| Lagelu | 0 (0, 37554) | 2196 (0, 34303) |
| LagosIsland | 0 (0, 39685) | 1336 (0, 75728) |
| Lamurde | 0 (0, 31788) | 2813 (0, 18610) |
| Langtang North | 0 (0, 37683) | 1804 (0, 30557) |
| Langtang South | 0 (0, 29958) | 2675 (0, 24789) |
| Lapai | 0 (0, 36794) | 7487 (607, 21421) |
| Lau | 0 (0, 28557) | 4369 (166, 18752) |
| Lavun | 0 (0, 96105) | 14806 (925, 51025) |
| Lere | 0 (0, 93814) | 20761 (1526, 77675) |
| Logo | 0 (0, 41530) | 7547 (3372, 21546) |
| Lokoja | 0 (0, 71155) | 18398 (2004, 47240) |
| Machina | 0 (0, 0) | 4 (0, 5304) |
| Madagali | 0 (0, 22878) | 706 (0, 14675) |
| Madobi | 0 (0, 34438) | 3206 (0, 39943) |
| Mafa | 0 (0, 0) | 716 (0, 12921) |
| Magama | 0 (0, 47509) | 3917 (164, 23364) |
| Magumeri | 0 (0, 44298) | 2646 (0, 20220) |
| Mai'Adua | 0 (0, 65969) | 0 (0, 54614) |
| Maidugur | 0 (0, 97900) | 0 (0, 79943) |
| Maigatari | 0 (0, 0) | 0 (0, 8431) |
| Maiha | 0 (0, 17899) | 1301 (0, 13365) |
| Mainland | 0 (0, 194628) | 0 (0, 202996) |
| Maiyama | 0 (0, 0) | 489 (0, 21312) |
| Makarfi | 0 (0, 37184) | 3695 (0, 33040) |
| Makoda | 0 (0, 46638) | 1016 (0, 28501) |
| Makurdi | 99457 (0, 99457) | 63009 (27598, 97657) |
| MalamMad | 0 (0, 0) | 0 (0, 5484) |
| Malumfashi | 0 (0, 55476) | 4985 (0, 68192) |
| Mangu | 0 (0, 86551) | 20709 (1476, 77899) |
| Mani | 0 (0, 52079) | 671 (0, 29760) |
| Maradun | 0 (0, 0) | 370 (0, 17136) |
| Mariga | 0 (0, 58081) | 13341 (1866, 37835) |
| Marte | 0 (0, 11947) | 414 (0, 19747) |
| Maru | 0 (0, 106795) | 23434 (6345, 52207) |
| Mashegu | 0 (0, 0) | 10806 (2186, 24745) |
| Mashi | 0 (0, 49632) | 239 (0, 34900) |
| Matazu | 0 (0, 30619) | 2909 (0, 25302) |
| Mayo-Bel | 0 (0, 50172) | 5018 (21, 33520) |
| Mbaitoli | 0 (0, 72920) | 29900 (0, 74317) |
| Mbo | 0 (0, 29413) | 2459 (0, 28928) |
| Michika | 0 (0, 31813) | 1760 (0, 22883) |
| Miga | 0 (0, 0) | 81 (0, 5547) |
| Mikang | 0 (0, 25026) | 1586 (0, 17822) |
| Minjibir | 0 (0, 64791) | 3791 (0, 60929) |
| Misau | 0 (0, 70375) | 1484 (0, 48786) |
| Mkpat Enin | 0 (0, 51560) | 15082 (0, 59288) |
| Moba | 0 (0, 29562) | 5783 (0, 37799) |
| Mobbar | 0 (0, 10519) | 1083 (0, 17006) |
| Mokwa | 0 (0, 65381) | 11789 (2224, 35116) |
| Monguno | 0 (0, 24145) | 155 (0, 13694) |
| Mopa-Muro | 14543 (0, 14543) | 6632 (148, 18513) |
| Moro | 33431 (0, 33431) | 15092 (6538, 23363) |
| Mubi North | 0 (0, 39128) | 2600 (0, 25617) |
| Mubi South | 0 (0, 15214) | 435 (0, 14399) |
| Musawa | 0 (0, 48269) | 7986 (0, 36909) |
| Mushin | 0 (0, 94710) | 0 (0, 113765) |
| Muya | 0 (0, 27978) | 5824 (297, 20060) |
| Nafada | 0 (0, 46110) | 1163 (0, 24084) |
| Nangere | 0 (0, 54214) | 24 (0, 44379) |
| Nasarawa | 0 (0, 58240) | 13201 (2352, 37624) |
| Nassaraw | 0 (0, 130739) | 0 (0, 130739) |
| Nassarawa Egon | 0 (0, 39790) | 6781 (0, 34561) |
| Ndokwa East | 0 (0, 34195) | 11796 (366, 31979) |
| Ndokwa West | 0 (0, 43841) | 15648 (0, 51750) |
| Nembe | 0 (0, 40047) | 10678 (64, 37873) |
| Ngala | 0 (0, 14310) | 23 (0, 22102) |
| Nganzai | 0 (0, 27793) | 618 (0, 15785) |
| Ngaski | 0 (0, 0) | 1388 (0, 11862) |
| Ngor-Okp | 48253 (0, 48253) | 21912 (0, 62127) |
| Nguru | 0 (0, 0) | 25 (0, 11655) |
| Ningi | 0 (0, 122948) | 18213 (3124, 63215) |
| Njaba | 0 (0, 47746) | 15286 (0, 68284) |
| Njikoka | 0 (0, 0) | 0 (0, 10346) |
| Nkanu East | 0 (0, 49990) | 9129 (22, 31755) |
| Nkanu West | 0 (0, 47895) | 5532 (0, 48956) |
| Nkwerre | 0 (0, 23857) | 1256 (0, 27323) |
| NnewiNort | 0 (0, 0) | 0 (0, 547) |
| NnewiSou | 0 (0, 0) | 2100 (0, 27904) |
| Nsit Atai | 0 (0, 42148) | 4342 (0, 33068) |
| Nsit Ibom | 0 (0, 23178) | 7237 (0, 27883) |
| Nsit Ubium | 0 (0, 33752) | 8928 (0, 44121) |
| Nsukka | 0 (0, 0) | 2633 (0, 15025) |
| Numan | 0 (0, 39529) | 378 (0, 27663) |
| Nwangele | 0 (0, 30632) | 3932 (0, 34257) |
| Obafemi-Owode | 0 (0, 81968) | 19288 (1645, 69802) |
| Obanliku | 40294 (0, 40294) | 18941 (958, 47804) |
| Obi | 19865 (0, 25328) | 11640 (34, 29304) |
| Obi | 0 (0, 41463) | 4417 (0, 33998) |
| Obio-Akp | 0 (0, 175444) | 44700 (0, 208582) |
| Obokun | 0 (0, 42089) | 10785 (0, 47202) |
| Oboma Ngwa | 0 (0, 78615) | 11673 (0, 92306) |
| Obot Akara | 0 (0, 36725) | 9271 (0, 45125) |
| Obowo | 0 (0, 29543) | 4672 (0, 43710) |
| Obubra | 0 (0, 55280) | 23238 (748, 61822) |
| Obudu | 15930 (0, 31860) | 13125 (0, 45647) |
| Odeda | 0 (0, 27865) | 9517 (1145, 25687) |
| Odigbo | 68098 (0, 68098) | 42495 (23434, 68181) |
| Odo0tin | 0 (0, 36001) | 5936 (0, 40917) |
| Odogbolu | 37673 (0, 37673) | 21113 (4285, 46403) |
| Odukpani | 0 (0, 41978) | 15051 (411, 40607) |
| Offa | 0 (0, 33014) | 219 (0, 28234) |
| Ofu | 0 (0, 50994) | 20598 (2358, 54022) |
| Ogbadibo | 0 (0, 48491) | 5915 (0, 45628) |
| Ogba-Egbe | 75954 (0, 75954) | 33154 (491, 82866) |
| Ogbaru | 0 (0, 25375) | 4054 (0, 16378) |
| Ogbia | 0 (0, 51229) | 18438 (0, 59262) |
| Ogbomosho North | 0 (0, 61084) | 5357 (0, 73311) |
| Ogbomosho South | 0 (0, 31308) | 2896 (0, 35136) |
| Ogoja | 0 (0, 46976) | 18280 (422, 52068) |
| Ogo-Oluw | 0 (0, 25726) | 10318 (339, 32878) |
| Ogori-Magongo | 10129 (0, 20258) | 5874 (0, 20188) |
| Ogu-Bolo | 0 (0, 6389) | 215 (0, 4657) |
| OgunWaterside | 0 (0, 24765) | 5945 (273, 22683) |
| Oguta | 44865 (0, 44865) | 25947 (1018, 51446) |
| Ohafia Abia | 0 (0, 78826) | 23351 (0, 87779) |
| Ohaji-Eg | 0 (0, 69183) | 26888 (2057, 68478) |
| Ohaozara | 0 (0, 39900) | 9777 (0, 45528) |
| Ohaukwu | 0 (0, 59605) | 9963 (0, 52643) |
| Ohimini | 24097 (0, 24097) | 12777 (744, 23258) |
| Oji-River | 0 (0, 38519) | 5396 (228, 24202) |
| Ojo | 0 (0, 166051) | 58145 (222, 215646) |
| Oju | 43206 (0, 43206) | 20285 (680, 60984) |
| Oke-Ero | 0 (0, 21629) | 7222 (1376, 15605) |
| Okehi | 0 (0, 88941) | 16346 (391, 89207) |
| Okene | 0 (0, 15774) | 5607 (0, 20064) |
| Okigwe | 0 (0, 38595) | 1538 (0, 48172) |
| Okitipupa | 68967 (0, 137933) | 70753 (20999, 113766) |
| Okobo | 0 (0, 29901) | 9452 (0, 32592) |
| Okpe | 0 (0, 37839) | 12343 (0, 43982) |
| Okpokwu | 0 (0, 43700) | 16900 (883, 31352) |
| Okrika | 0 (0, 50696) | 6049 (0, 44467) |
| Olamabor | 0 (0, 55392) | 18367 (627, 56886) |
| Ola-Oluwa | 0 (0, 23526) | 6590 (0, 32342) |
| Olorunda | 0 (0, 60656) | 1455 (0, 66678) |
| Olorunsogo | 23481 (0, 46963) | 15875 (1058, 36954) |
| Oluyole | 0 (0, 79884) | 18651 (452, 70937) |
| Omala | 0 (0, 32817) | 13189 (877, 34134) |
| Omumma | 0 (0, 33933) | 11876 (0, 47133) |
| Ona-Ara | 0 (0, 102034) | 9087 (0, 96469) |
| Ondo East | 0 (0, 15723) | 3477 (387, 15935) |
| Ondo West | 0 (0, 46426) | 1350 (0, 42125) |
| Onicha | 0 (0, 59399) | 14763 (0, 73656) |
| Onitsha North | 0 (0, 0) | 0 (0, 4003) |
| Onitsha South | 0 (0, 3146) | 0 (0, 2145) |
| Onna | 0 (0, 65653) | 3606 (0, 52431) |
| Opobo-Nkoro | 0 (0, 0) | 984 (0, 18715) |
| Oredo Edo | 0 (0, 57266) | 5228 (0, 60614) |
| Orelope | 0 (0, 30411) | 6990 (60, 36842) |
| Orhionmw | 0 (0, 55337) | 23718 (2480, 66112) |
| Oriade | 0 (0, 54449) | 5289 (0, 44204) |
| Ori-Ire | 39470 (0, 39470) | 27402 (9378, 56012) |
| Orlu | 0 (0, 52338) | 5823 (0, 72035) |
| Orolu | 0 (0, 85411) | 3983 (0, 79392) |
| Oron | 0 (0, 31269) | 820 (0, 37519) |
| Orsu | 0 (0, 53023) | 4266 (0, 56601) |
| Oru East | 0 (0, 37920) | 14687 (0, 48344) |
| Oru West | 36877 (0, 36877) | 21710 (0, 41825) |
| Oruk-Ana | 0 (0, 44886) | 13900 (0, 50946) |
| OrumbaNo | 0 (0, 22312) | 5425 (0, 34681) |
| OrumbaSo | 0 (0, 52444) | 2635 (0, 57074) |
| Ose | 44474 (0, 44474) | 25080 (3720, 49321) |
| Oshimili North | 0 (0, 30905) | 7329 (0, 34901) |
| Oshimili South | 0 (0, 46978) | 2881 (0, 42849) |
| Oshodi-Isolo | 0 (0, 134315) | 0 (0, 166831) |
| Osisioma Ngwa | 0 (0, 66146) | 7539 (0, 88228) |
| Osogbo | 0 (0, 41480) | 1170 (0, 35808) |
| Oturkpo | 0 (0, 84921) | 19525 (5507, 36519) |
| OviaNort | 46135 (0, 46135) | 21942 (5964, 54770) |
| OviaSouth-West | 48349 (0, 48349) | 27384 (6324, 56979) |
| Owan East | 0 (0, 44446) | 18985 (1145, 44877) |
| OwanWest | 27836 (0, 27836) | 11466 (466, 29300) |
| Owerri Municipal | 0 (0, 35192) | 0 (0, 35308) |
| Owerri North | 0 (0, 44327) | 12106 (0, 53423) |
| Owerri West | 0 (0, 38951) | 12032 (0, 41831) |
| Owo | 63512 (63512, 63512) | 52161 (38411, 69360) |
| Oye | 40735 (0, 81470) | 19343 (0, 63344) |
| Oyi | 0 (0, 0) | 177 (0, 17079) |
| Oyigbo | 0 (0, 50493) | 13256 (0, 64501) |
| Oyo East | 0 (0, 39697) | 2905 (0, 46625) |
| Oyo West | 0 (0, 43801) | 1079 (0, 42262) |
| Oyun | 0 (0, 27853) | 4610 (666, 13349) |
| Paikoro | 0 (0, 47695) | 7967 (591, 35040) |
| Pankshin | 0 (0, 46944) | 11538 (409, 46797) |
| Patani | 18242 (0, 18242) | 9051 (0, 25609) |
| Pategi | 0 (0, 32914) | 5890 (365, 17397) |
| Port Harcourt | 0 (0, 167344) | 7597 (0, 118980) |
| Potiskum | 0 (0, 35295) | 0 (0, 24266) |
| Qua'anpa | 0 (0, 52923) | 7435 (78, 38120) |
| Rabah | 0 (0, 0) | 168 (0, 5366) |
| Rafi | 0 (0, 50677) | 9674 (1660, 31406) |
| Rano | 0 (0, 34918) | 4327 (0, 35798) |
| Remo-North | 0 (0, 31961) | 3421 (0, 28879) |
| Rijau | 0 (0, 49413) | 4908 (127, 24765) |
| Rimi | 0 (0, 50883) | 35 (0, 30588) |
| RiminGad | 0 (0, 26358) | 1444 (0, 31442) |
| Ringim | 0 (0, 57558) | 2766 (0, 44102) |
| Riyom | 0 (0, 36713) | 8519 (0, 35988) |
| Rogo | 0 (0, 66587) | 9190 (0, 54152) |
| Roni | 0 (0, 14617) | 64 (0, 10120) |
| Sabon Birni | 0 (0, 0) | 462 (0, 8554) |
| Sabon-Ga | 0 (0, 107443) | 2655 (0, 101705) |
| Sabuwa | 0 (0, 36107) | 5250 (0, 35958) |
| Safana | 0 (0, 40348) | 2871 (0, 22234) |
| Sagbama | 50289 (0, 50289) | 22678 (811, 59328) |
| Sakaba | 0 (0, 27875) | 3848 (0, 21428) |
| Saki East | 0 (0, 75074) | 19695 (1796, 79899) |
| Saki West | 0 (0, 44434) | 17757 (1627, 51556) |
| Sandamu | 0 (0, 51441) | 0 (0, 39723) |
| Sanga | 0 (0, 42365) | 10170 (147, 38761) |
| Sapele | 0 (0, 52043) | 2379 (0, 63158) |
| Sardauna | 63097 (0, 63097) | 42332 (22049, 67658) |
| Shagamu | 0 (0, 73162) | 18065 (50, 85804) |
| Shagari | 0 (0, 0) | 2074 (0, 11773) |
| Shanga | 0 (0, 29798) | 1821 (0, 19059) |
| Shani | 0 (0, 30166) | 1576 (0, 21288) |
| Shanono | 0 (0, 41360) | 6476 (0, 39471) |
| Shelleng | 0 (0, 37311) | 4159 (8, 19762) |
| Shendam | 0 (0, 60153) | 6600 (0, 35152) |
| Shinkafi | 0 (0, 0) | 44 (0, 5730) |
| Shira | 0 (0, 50674) | 102 (0, 24862) |
| Shiroro | 0 (0, 72071) | 14793 (4088, 40114) |
| Shomgom | 0 (0, 48426) | 1910 (0, 32713) |
| Shomolu | 0 (0, 150988) | 0 (0, 157400) |
| Silame | 0 (0, 0) | 0 (0, 0) |
| Soba | 0 (0, 80271) | 16736 (105, 56697) |
| Sokoto North | 0 (0, 0) | 0 (0, 0) |
| Sokoto South | 0 (0, 0) | 0 (0, 360) |
| Song | 0 (0, 60986) | 6403 (473, 34338) |
| Southern Ijaw | 0 (0, 93663) | 27138 (4859, 66528) |
| Suleja | 0 (0, 36323) | 270 (0, 42265) |
| Sule-Tan | 0 (0, 0) | 1118 (0, 27072) |
| Sumaila | 0 (0, 65629) | 8948 (0, 45113) |
| Suru | 0 (0, 10856) | 589 (0, 18462) |
| Surulere | 0 (0, 245731) | 0 (0, 246429) |
| Surulere | 42478 (0, 42478) | 22064 (1804, 49564) |
| Tafa | 0 (0, 41388) | 1639 (0, 49245) |
| Tafawa-B | 0 (0, 56561) | 10475 (654, 39309) |
| Tai | 0 (0, 27241) | 4242 (0, 33888) |
| Takai | 0 (0, 70061) | 3792 (0, 42686) |
| Takum | 23615 (0, 23615) | 10640 (1296, 22350) |
| Talata-Mafara | 0 (0, 0) | 13 (0, 5521) |
| Tambawal | 0 (0, 0) | 4757 (0, 26882) |
| Tangazar | 0 (0, 0) | 0 (0, 4260) |
| Tarauni | 0 (0, 95613) | 0 (0, 95338) |
| Tarka | 16328 (0, 20818) | 8495 (18, 25106) |
| Tarmuwa | 0 (0, 5906) | 930 (0, 9535) |
| Taura | 0 (0, 31657) | 1237 (0, 14705) |
| Teungo | 14286 (0, 14286) | 6635 (1654, 14624) |
| Tofa | 0 (0, 24301) | 1519 (0, 25341) |
| Toro | 0 (0, 107166) | 30854 (6331, 61171) |
| Toto | 0 (0, 32247) | 7068 (472, 28496) |
| Tsafe | 0 (0, 78546) | 12975 (1427, 38202) |
| Tsanyawa | 0 (0, 28090) | 768 (0, 23411) |
| Tundun Wada | 0 (0, 61934) | 12430 (0, 46276) |
| Tureta | 0 (0, 11002) | 1372 (0, 6205) |
| Udenu | 0 (0, 38217) | 2203 (0, 30224) |
| Udi | 0 (0, 72543) | 16533 (755, 53152) |
| Udu | 0 (0, 49500) | 11388 (0, 69734) |
| Udung Uko | 0 (0, 14163) | 109 (0, 11924) |
| Ughelli North | 0 (0, 99206) | 25633 (1786, 115843) |
| Ughelli South | 0 (0, 74347) | 22136 (566, 84475) |
| Ugwunagbo | 0 (0, 20575) | 8432 (0, 26621) |
| Uhunmwonde | 0 (0, 57151) | 21116 (2440, 55194) |
| Ukanafun | 0 (0, 39709) | 11992 (0, 49279) |
| Ukum | 60914 (0, 60914) | 32183 (2755, 60076) |
| Ukwa East | 0 (0, 13781) | 5460 (0, 17147) |
| Ukwa West | 0 (0, 37983) | 10220 (0, 49829) |
| Ukwuani | 0 (0, 34525) | 9911 (0, 38482) |
| Umuahia North | 0 (0, 210522) | 17899 (0, 168744) |
| Umuahia South | 0 (0, 42921) | 6890 (0, 31415) |
| Umu-Nneochi | 0 (0, 47706) | 7840 (0, 49397) |
| Ungogo | 0 (0, 164655) | 133 (0, 144824) |
| Unuimo | 0 (0, 22057) | 3942 (0, 29948) |
| Uruan | 0 (0, 37917) | 12139 (0, 41219) |
| UrueOffo | 0 (0, 37268) | 1932 (0, 29393) |
| Ushongo | 0 (0, 70976) | 22156 (3342, 57372) |
| Ussa | 39810 (0, 39810) | 24385 (8328, 44394) |
| Uvwie | 0 (0, 62970) | 1492 (0, 71371) |
| Uyo | 0 (0, 85156) | 15280 (0, 109078) |
| Uzo-Uwani | 0 (0, 0) | 3194 (94, 11574) |
| Vandeiky | 0 (0, 51853) | 15801 (0, 59777) |
| Wamakko | 0 (0, 0) | 0 (0, 38) |
| Wamba | 0 (0, 21128) | 5520 (12, 23376) |
| Warawa | 0 (0, 65854) | 4029 (0, 54443) |
| Warji | 0 (0, 0) | 16 (0, 12440) |
| Warri North | 0 (0, 35531) | 10592 (980, 32623) |
| Warri South | 0 (0, 81193) | 15150 (0, 95076) |
| Warri South-West | 0 (0, 29590) | 9424 (704, 25289) |
| Wase | 0 (0, 67111) | 6693 (378, 39095) |
| Wudil | 0 (0, 64249) | 3620 (0, 70887) |
| Wukari | 0 (0, 69212) | 10665 (2176, 40859) |
| Wurno | 0 (0, 0) | 1483 (0, 13598) |
| Wushishi | 0 (0, 24537) | 2714 (0, 21660) |
| Yabo | 0 (0, 0) | 0 (0, 2327) |
| Yagba East | 0 (0, 44805) | 16182 (1562, 49506) |
| Yagba West | 0 (0, 38688) | 8385 (391, 32780) |
| Yakurr | 0 (0, 55969) | 19317 (3, 73697) |
| Yala Cross | 0 (0, 63528) | 24600 (3002, 65325) |
| Yamaltu | 0 (0, 19556) | 1778 (0, 35825) |
| Yankwashi | 0 (0, 36372) | 2639 (0, 20189) |
| Yauri | 0 (0, 0) | 879 (0, 7909) |
| Yenegoa | 0 (0, 95215) | 26319 (366, 124991) |
| Yola North | 0 (0, 29844) | 0 (0, 23619) |
| Yola South | 0 (0, 31273) | 0 (0, 17969) |
| Yorro | 0 (0, 36311) | 11788 (1127, 37237) |
| Yunusari | 0 (0, 0) | 2098 (0, 15079) |
| Yusufari | 0 (0, 0) | 1510 (0, 10688) |
| Zaki | 0 (0, 0) | 635 (0, 19945) |
| Zango | 0 (0, 51212) | 226 (0, 27460) |
| ZangonKa | 0 (0, 107729) | 25085 (2468, 108192) |
| Zaria | 0 (0, 103495) | 3015 (0, 114519) |
| Zing | 0 (0, 26782) | 3635 (0, 25550) |
| Zurmi | 0 (0, 0) | 1084 (0, 9338) |
| Zuru | 0 (0, 38818) | 1910 (0, 36564) |
| Total* | 9025229 (6239358, 12153254) | 10222409 (7702724, 13045014) |

*Of note: the total treatments are based on summing all LGA-specific treatment distributions, and not summaries (e.g. medians) of each. This is due to the fact that sum of summaries do not correspond to summaries of sums of distributions.
