# Supplementary material for: Bayesian Geostatistical Model-Based Estimates of Soil-Transmitted Helminth Infection in Nigeria, Including Annual Deworming Requirements
Source: PLoS Negl Trop Dis. 2015 Apr 24;9(4):e0003740. doi: 10.1371/journal.pntd.0003740 (PMC4409219; doi:10.1371/journal.pntd.0003740)
Supplement: S1 Text — (DOCX) [file pntd.0003740.s004.docx]

**Details of Analysis conducted**

We applied Bayesian binomial geostatistical models to relate STH infection risk with environmental and socioeconomic predictors. We used integrated nested Laplace approximations (INLA) [1] and the stochastic partial differential equations (SPDE) approach [2] for fast approximate Bayesian inference. Analysis was carried out in R [3] and with the INLA package (available at [www.r-inla.org](http://www.r-inla.org)).

In brief, the Bayesian binomial geostatistical model assumes that (for example) hookworm positives Y_i_ at location s_i_ arise from a binomial distribution Bin(p_i_,n_i_), where p_i_ is the infection prevalence and n_i_ is the number of examined children at location s_i_.The logit of p_i_is then logit(p_i_)=X^T^_i_**β**+w_i_, where X_i_ is the vector of covariates (including an intercept) observed at location s_i_, **β** is the respective coefficient vector and w_i_ is a random intercept. It is assumed that **w** (the vector of all w_i_) is a realization of a Gaussian process and thus**w**~MVN(0,Σ(**θ**)).Correlation in space is taken into account through the spatially structured covariance matrix Σ of **w** which depends on the hyperparameters**θ**(i.e. range and variance). A Matérncovariance function and the SPDE approach was used[2,4,5].The Bayesian model formulation is completed by assigning normal prior distributions to **β** and **θ** (at a transformed scale). The INLA inferential approach is based on the Gaussian approximation of the full conditional posterior distribution of the Gaussian field which is constituted by **β** and **w**. The marginal posterior distribution of **θ** is then approximated with a Laplace approximation. For more details the reader is referred to[1].

In geostatistical disease mapping, model selection, i.e. which predictors constitute **X**_i_, have been performed through numerous approaches [6]. We followed the approach by Karagiannis-Voules et al. [7].In particular, we chose the functional form of each predictor with the best logarithmic score in bivariate models. We considered linear and categorical forms. Non-linearity was also addressed through random walk processes of order 1 and 2 [8]. Then, to identify the set (one for each species) of most important predictors, we fitted geostatistical models with all possible combinations of covariates, with their corresponding functional form found before, and selected the one with the best mean logarithmic score. The leave-one-out cross-validated logarithmic score [9,10] was used in all comparisons to select between models. These final Bayesian geostatistical models (one for each species) were used to predict infection risk at a grid of 3×3km.

**References**

[1] Rue H., Martino S. and Chopin N., 2009, Approximate Bayesian inference for latent Gaussian models using integrated nested Laplace approximations. Journal of the Royal Statistical Society, Series B (Statistical Methodology), 71, 319–392.

[2] Lindgren, F., Rue, H. and Lindström, J., 2011, An explicit link between Gaussian fields and Gaussian Markov random fields: the stochastic partial differential equation approach. Journal of the Royal Statistical Society, Series B (Statistical Methodology), 73: 423–498.

[3] R Core Team, 2014, R: A language and environment for statistical computing. R Foundation for Statistical Computing: Vienna, Austria, <http://www.R-project.org>.

[4] Whittle, P., 1954,On stationary processes in the plane. Biometrika, 41: 434–449.

[5] Whittle, P., 1963, Stochastic processes in several dimensions. Bulletin of the International Statistical Institute, 40: 974–994.

[6] Chammartin, F., Hürlimann E., Raso, G., et al., 2013,Statistical methodological issues in mapping historical schistosomiasis survey data. ActaTropica, 128: 345-352.

[7] Karagiannis-Voules, D.A., Biedermann, P., Ekpo, U.F., et al., 2015, Spatial and temporal distribution of soil-transmitted helminth infection in sub-Saharan Africa: a systematic review and geostatistical meta-analysis. The Lancet Infectious Diseases, 15: 74-84.

[8] Rue, H., and Held, L., 2005, Gaussian Markov random fields: theory and applications. Chapman & Hall/CRC, Boca Raton.

[9] Gneiting T., andRaftery A.E., 2007,Strictly proper scoring rules, prediction, and estimation. Journal of the American Statistical Association, 102:359-378.

[10] Held, L., Schrödle, B., and Rue, H., 2010, Posterior and cross-validatory predictive checks: a comparison of MCMC and INLA. In: Tutz G, Kneib T, eds. Statistical modelling and regression structures - Festschrift in honour of Ludwig Fahrmeir. Heidelberg, Dordrecht, London, New York: Springer.
